# Influenza H3N2 infection of the collaborative cross founder strains reveals highly divergent host responses and identifies a unique phenotype in CAST/EiJ mice

**DOI:** 10.1186/s12864-016-2483-y

**Published:** 2016-02-27

**Authors:** Sarah R. Leist, Carolin Pilzner, Judith M.A. van den Brand, Leonie Dengler, Robert Geffers, Thijs Kuiken, Rudi Balling, Heike Kollmus, Klaus Schughart

**Affiliations:** Department of Infection Genetics, Helmholtz Centre for Infection Research, Braunschweig and University of Veterinary Medicine Hannover, Inhoffenstr.7, D-38124 Braunschweig, Hannover Germany; Department of Viroscience, Erasmus Medical Center, Rotterdam, Netherlands; Genome Analytics, Helmholtz Centre for Infection Research, Braunschweig, Germany; Luxembourg Centre for Systems Biomedicine (LCSB), University of Luxembourg, Esch-sur-Alzette, Luxembourg; University of Tennessee Health Science Center, Memphis, TN USA

**Keywords:** Influenza susceptibility, Collaborative cross, Phenotyping, Mouse model, Immune response

## Abstract

**Background:**

Influenza A virus is a zoonotic pathogen that poses a major threat to human and animal health. The severe course of influenza infection is not only influenced by viral virulence factors but also by individual differences in the host response. To determine the extent to which the genetic background can modulate severity of an infection, we studied the host responses to influenza infections in the eight genetically highly diverse Collaborative Cross (CC) founder mouse strains.

**Results:**

We observed highly divergent host responses between the CC founder strains with respect to survival, body weight loss, hematological parameters in the blood, relative lung weight and viral load. Mouse strain was the main factor with highest effect size on body weight loss after infection, demonstrating that this phenotype was highly heritable. Sex represented another significant main effect, although it was less strong. Analysis of survival rates and mean time to death suggested three groups of susceptibility phenotypes: highly susceptible (A/J, CAST/EiJ, WSB/EiJ), intermediate susceptible (C57BL/6J, 129S1/SvImJ, NOD/ShiLtJ) and highly resistant strains (NZO/HlLtJ, PWK/PhJ). These three susceptibility groups were significantly different with respect to death/survival counts. Viral load was significantly different between susceptible and resistant strains but not between intermediate and highly susceptible strains. CAST/EiJ mice showed a unique phenotype. Despite high viral loads in their lungs, CAST/EiJ mice exhibited low counts of infiltrating granulocytes and showed increased numbers of macrophages in the lung. Histological studies of infected lungs and transcriptome analyses of peripheral blood cells and lungs confirmed an abnormal response in the leukocyte recruitment in CAST/EiJ mice.

**Conclusions:**

The eight CC founder strains exhibited a large diversity in their response to influenza infections. Therefore, the CC will represent an ideal mouse genetic reference population to study the influence of genetic variation on the susceptibility and resistance to influenza infections which will be important to understand individual variations of disease severity in humans. The unique phenotype combination in the CAST/EiJ strain resembles human leukocyte adhesion deficiency and may thus represent a new mouse model to understand this and related abnormal immune responses to infections in humans.

**Electronic supplementary material:**

The online version of this article (doi:10.1186/s12864-016-2483-y) contains supplementary material, which is available to authorized users.

## Background

Every year, about 500,000 people die world-wide from influenza virus infections [1]. In recent history, the emergence of new influenza subtypes has caused several pandemics [2–4]. The most severe pandemic in 1918 resulted in about 30–50 million deaths worldwide [5] and a new variant of a seasonal H1N1 virus, pH1N1, caused a world-wide pandemic in 2009 [6, 7]. The course and outcome of an infection is determined by pathogen virulence, environmental and host factors. Genetic diversity of the host has been found as an important factor that strongly influences the host response and severity to influenza infections in humans [8–12]. Environmental and genetic factors can be well controlled in experimental animal models. It has been shown in mouse models that the genetic make-up of the host strongly influences the disease outcome after influenza infections (reviewed in [11, 13]). Subsequently, several genes have been described also in humans that contribute to disease severity [14].

Mouse genetic reference populations (GRPs) have become an important experimental system that allows investigating the influence of genetic variation on human diseases (reviewed in [15–21]. In addition, GRPs have been used to study the influence of genetic variation on the host response to infectious pathogens (reviewed in [13, 22–24]. The Collaborative Cross (CC) is a recently created population that has been generated from eight inbred mouse strains, referred to as founder [25]. The addition of three wild-derived strains (CAST/EiJ, PWK/PhJ and WSB/EiJ) strongly enhanced the genetic diversity of the CC [26]. Several studies in not fully inbred CC strains (pre-CC strains) and CC strains themselves led to the identification of quantitative trait loci (QTLs) influencing human diseases [27–31]. More recently, CC F1 hybrids have been identified that exhibited severe hemorrhagic fever, similar to humans, after infection with Ebola virus [32]. Another CC strain has been shown to represent a new model for spontaneous colitis [33].

Here, we investigated the pathology, disease severity and the host response in the eight CC founder strains to H3N2 influenza A virus infections. We observed a broad range of phenotypic outcomes for many pathological and molecular parameters. Also, we found a specific defect in highly susceptible CAST/EiJ mice that resembled leukocyte recruitment deficiency in humans.

## Results

### Three different groups of susceptibility phenotypes are observed after infection of CC founder strains with H3N2 virus

**Fig. 1:**
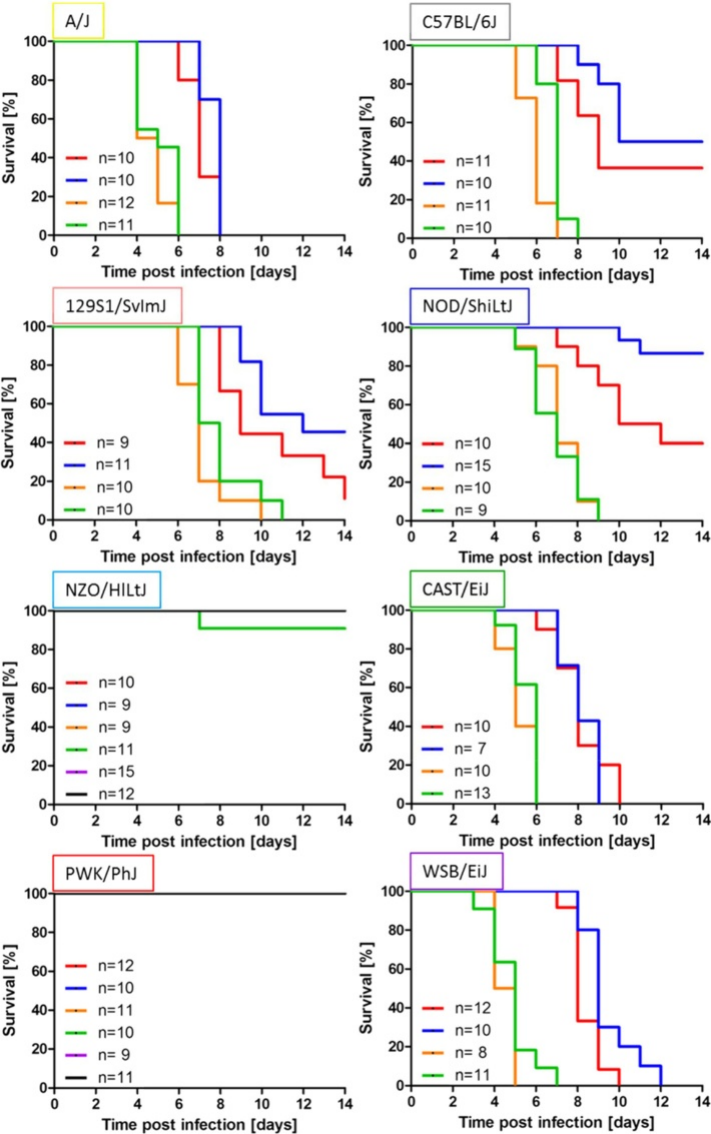
Survival rates of the eight CC founder strains after infection with different doses of influenza A H3N2 virus infection. Eight to twelve weeks old female and male mice of the CC founder strains were infected intra-nasally with 1 × 10^1^ (red and blue), 2 × 10^3^ (orange and green) and 2 × 10^5^ FFU (magenta and black) of the mouse-adapted influenza H3N2 virus (A/HK/01/68). Survival rates were monitored for 14 days p.i.. Mice that exhibited a weight loss of more than 30 % relative to their starting weight were euthanized and scored as dead. Significant sex-specific differences in survival curves were observed exclusively in NOD/ShiLtJ mice after infection with 1 × 10^1^ FFU (**: p-value < 0.01, using log rank test). Colors for the CC founder strains have been chosen according to the CC strain RGB color code displayed at http://csbio.unc.edu/CCstatus/index.py.

Eight to twelve weeks old female and male mice of the eight founder strains were infected intra-nasally with a dose of 1 × 10^1^ FFU of the mouse-adapted influenza virus subtype H3N2 (A/HK/01/68). Survival rates of infected mice were monitored for a period of 14 days (Fig. 1). Our results suggest three different susceptibility groups based on survival rates and mean time to death: Highly susceptible, intermediate susceptible and highly resistant strains. Pair-wise chi-square tests between the three groups revealed significant differences for the death/survival counts (Additional file 1: Table S1). The strains A/J, CAST/EiJ and WSB/EiJ belonged to the highly susceptible group in which all infected mice died. NZO/HlLtJ and PWK/PhJ were found to be highly resistant; all infected mice survived the infection. Three strains, C57BL/6J, 129S1/SvImJ and NOD/ShiLtJ, showed intermediate susceptibility with survival rates ranging from 10 % to 90 %. No significant differences were observed in the survival curves of male and female mice for all strains except for NOD/ShiLtJ, whereby females were more susceptible than males. At an increased infection dose of 2 × 10^3^ FFU all mice from highly and intermediate susceptible strains (A/J, C57BL/6J, 129S1/SvImJ, NOD/ShiLtJ, CAST/EiJ and WSB/EiJ) succumbed to the infection (Fig. 1). In contrast, the two highly resistant strains (NZO/HlLtJ and PWK/PhJ) were also resistant to infections with higher infection doses of 2 × 10^3^ FFU and 2 × 10^5^ FFU (Fig. 1). For all strains, except the highly resistant ones, survival rates in females and males at the lower dose were significantly higher than survival rates at higher doses (Fig. 1).

**Fig. 2:**
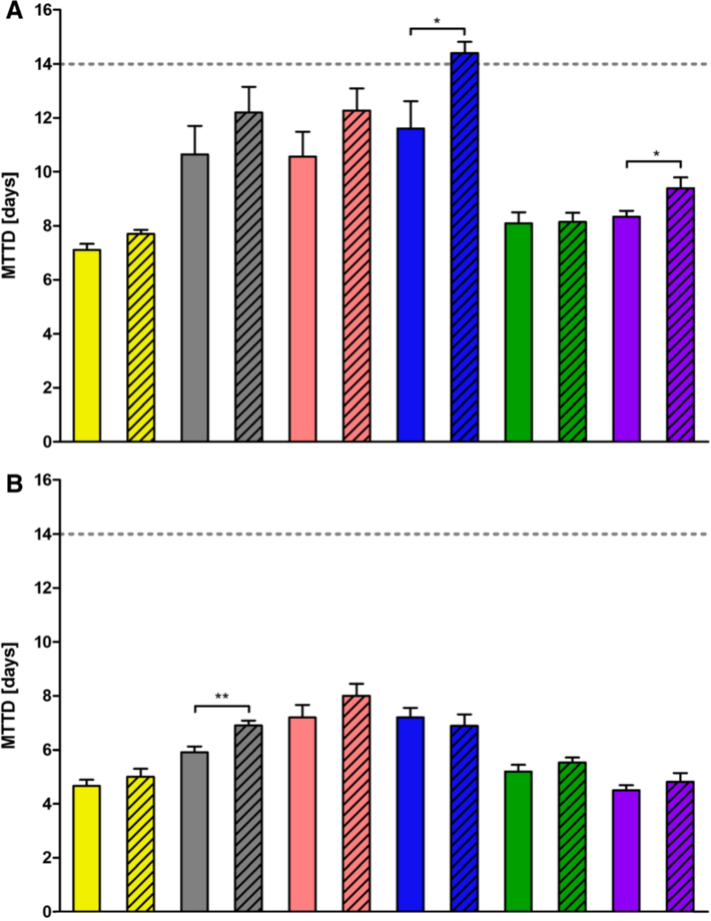
Sex-specific differences in mean time to death at different doses of influenza A H3N2 virus infection in C57BL/6J, NOD/ShiLtJ and WSB/EiJ. The same data set and color code as in Fig. 1 was used to analyze mean time to death (MTTD) of the strains (clear bars: female; dashed bars: male mice). Changes in MTTD were calculated for 14 days p.i., survival was scored as an MTTD value of 15. Significant sex-specific differences were calculated for infection with 1 × 10^1^ FFU (**a**) and 2x10^3^ FFU (**b**). NOD/ShiLtJ and WSB/EiJ exhibited significant sex-specific differences in infections with 1 × 10^1^ FFU and C57BL/6J in infections with 2 × 10^3^ FFU. The dashed line marks the end of experiment. Significances were calculated using Mann Whitney *U* test and are marked by stars (*: *p* < 0.1; **: *p* < 0.01)

Next, we analyzed mean time to death (MTTD) to investigate how rapidly susceptible mice succumbed to infection. At an infection dose of 1 × 10^1^ FFU, the highly susceptible strains exhibited the lowest MTTD. The MTTD was 7 and 9 days p.i. whereas the intermediate susceptible strains died at day 11 to 14 p.i. (Fig. 2a). At higher infection dose (2 × 10^3^ FFU) the highly susceptible (day 5 to 6) and the intermediate susceptible group (day 7 to 8) had a lower MTTD (Fig. 2b). When comparing male and female mice, a significant difference of MTTD was found for NOD/ShiLtJ and WSB/EiJ mice at the low virus dose whereas at the high infection dose, only C57BL/6J mice exhibited a sex-dependent difference in MTTD (Fig. 2).

### Infection of CC founder strains leads to highly variable body weight changes

**Fig. 3:**
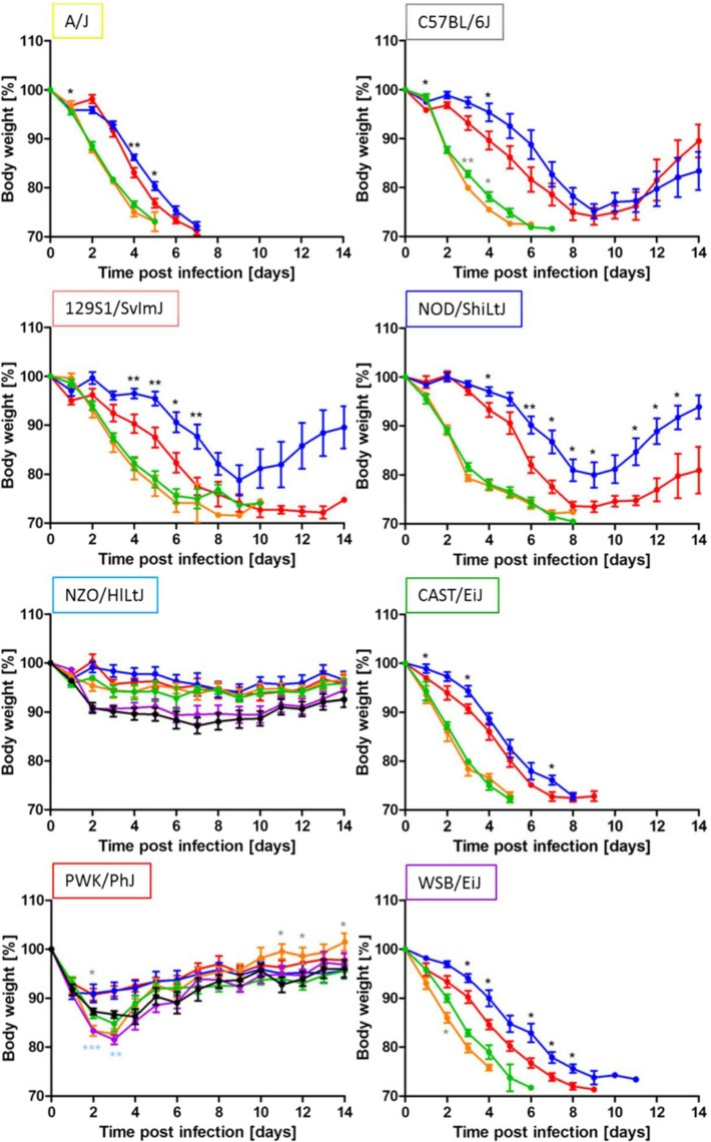
Dose-dependent differences in body weight changes after influenza A H3N2 virus infection. The same data set and color code as in Fig. 1 was used. Changes in body weight were monitored for 14 days p.i.. Significant sex-specific differences were observed in all CC founder strains and were calculated by Mann Whitney *U* test (*: *p* < 0.1; **: *p* < 0.01; ***: *p* < 0.001; black asterisk: 1 × 10^1^ FFU; grey asterisk: 2 × 10^3^ FFU; blue asterisk: 2 × 10^5^ FFU)

In a previous study [34], body weight changes in CC mice were recorded daily until day 4 after infection with H1N1 (A/PR/8/34 (PR8)) virus. In our studies, we aimed to provide a more complete phenotype description after H3N2 infection that covers all phases of the host response (innate, adaptive and recovery). Therefore, we measured body weight changes at each day after infection until recovery of body weight after 14 days p.i.. We also included both sexes in our study. The analysis of body weight changes over 14 days confirmed a large phenotypic diversity between susceptible and resistant strains and also within susceptible and resistant groups (Fig. 3). In addition, the resistant strains were infected with a dose of 2 × 10^5^ FFU. In this case, NZO/HlLtJ mice showed a drop in body weight (10 %) at day 2 which stayed low until day 14 after infection whereas PWK/PhJ showed higher body weight loss (10-20 %) at early days after infection and rapidly regained weight from day 4 on (Fig. 3).

A multi-factorial analysis of variance (ANOVA) was performed to investigate variation of body weight change as response to infection for the factors strains, sex and days. The analysis was limited to days 1 to 7 p.i. because for some strains (A/J, CAST/EiJ, and WSB/EiJ), more than 60 % of infected mice died by day 8. Thus, the surviving mice were not any more representative for the group. ANOVA (model: body weight loss ~ strain * sex * day and deleting the non-significant interaction strain:day:sex) revealed that all main effects (strain, sex and day) and all pair-wise interactions were significant (Additional file 2: Table S2). Subsequent Tukey-HSD post-hoc test was used to determine significance of pairs (strain by day p.i.; Additional file 3: Table S3). The changes in body weight were most pronounced on day 7 p.i.. Strains A/J, CAST/EiJ, and WSB/EiJ exhibited the highest loss of body weight whereas NZO/HlLtJ was least affected. The broad-sense heritability was determined for each day separately and ranged from 0.28 to 0.65 (Table 1) indicating high heritability of this trait. The sex-effect (using the optimized AVOVA model) was significant from days 3 to 7 p.i. but with a lower effect size (maximum of 8 %, Table 1).

**Table 1:** ANOVA-Broad-sense heritability and sex effect per day

Days p.i.	Heritability H^2^	Sex effect	*P*-value sex effect
day1	0.28	ns	ns
day2	0.39	ns	ns
day3	0.28	0.08	1.91e-05
day4	0.45	0.08	6.38e-07
day5	0.58	0.06	3.03e-06
day6	0.59	0.06	4.13e-07
day7	0.65	0.04	2.9e-05

*ns:* not significant, H^2^ broadsense heritability, Sex effect sum of squares for sex divided by total sum of squares using two-way ANOVA model: BWL ~ strain * sex for each day separately

Comparisons for all strains over 14 days p.i. (Fig. 3) confirmed that female mice from the highly and intermediate susceptible group exhibited a higher weight loss than male mice at the lower infection dose of 1 × 10^1^ FFU (Fig. 3). For the resistant strains NZO/HlLtJ and PWK/PhJ no differences between sexes were observed (Fig. 3). In contrast, at the higher infection dose (2 × 10^3^ FFU) no significant differences were observed between males and females except for some single days for C57BL/6J on day 3 and 4, PWK/PhJ on days 2, 11, 12 and 14 and WSB/EiJ on day 2 (Fig. 3). Male mice generally showed less severe changes in body weight than females. Only for the PWK/PhJ strain female mice exhibited significantly less body weight loss than male mice. However, this was only observed late after infection for days 11, 12 and 14.

### Selection of infection dose and gender for further experiments

For all following studies, we decided to perform infections with a dose of 1 × 10^1^ FFU because at this dose, variation between the founder strains was highest for survival, MTTD and body weight changes. Also, we limited the following analyses to female mice. Females were used extensively in our and other laboratories studies thus allowing better comparability to existing data than males. In addition, the sex effect was relatively small (ANOVA) compared to the strain effect. Also, in this study, we mainly focused on effects caused by genetic variation which is represented by the strain effect (heritability). Therefore, the additional information gained by including males would have been rather limited in our studies and, with respect to the 3R principle, was omitted.

### Increase in relative lung weight differs among CC founder strains

**Fig. 4:**
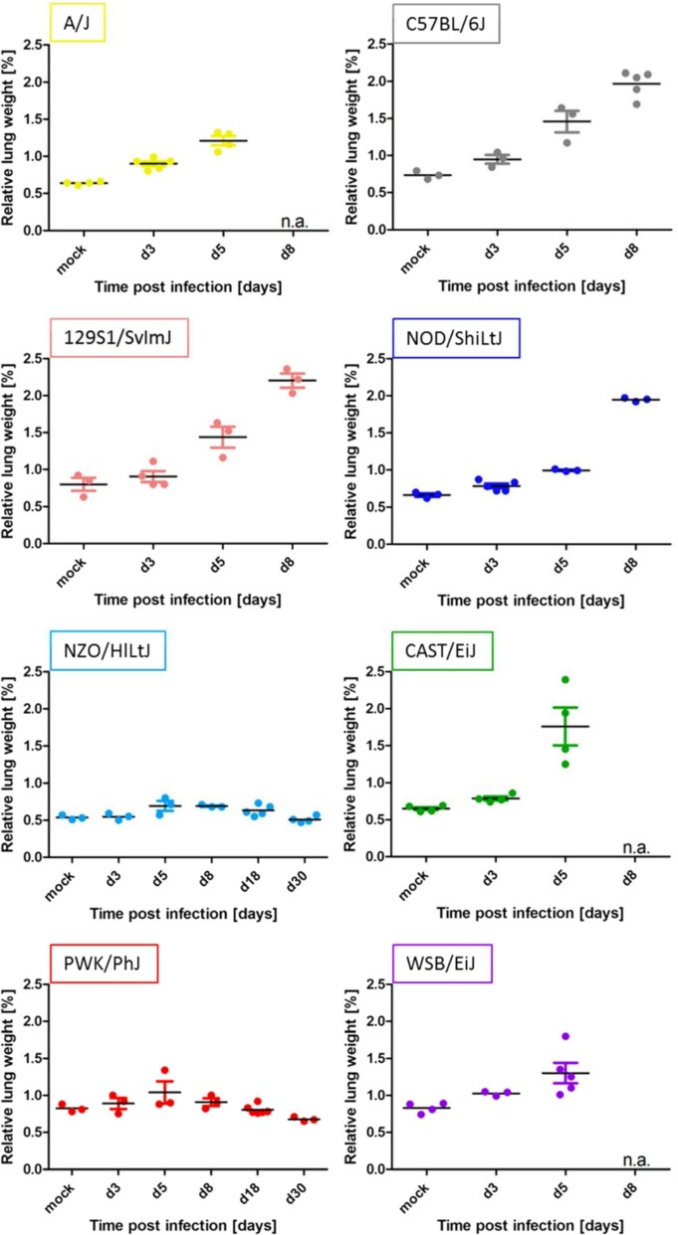
Increase in relative lung weight after influenza A H3N2 infection. Eight to twelve weeks old female mice of the eight CC founder strains were infected intra-nasally with 1 × 10^1^ FFU of the mouse-adapted influenza H3N2 virus (A/HK/01/68). Lung weight was determined on day 3, 5, 8, 18 and 30 p.i. and for mock infected animals on day 3 post PBS treatment. CC founder strains showed comparable increase in relative lung weight up to 2 % except for the highly resistant NZO/HlLtJ and PWK/PhJ. Each data point represents the measurement of one animal. Additionally, the mean and the standard error of the mean are shown for each time point and strain

The relative lung weight (wet lung weight relative to body weight on day of preparation) was investigated at different days after infection as measurement for the degree of immune cell infiltration and fluid influx into the lung (Fig. 4). For highly susceptible strains the relative lung weight increased significantly on day 5 p.i.. Lung weight increased for intermediate susceptible strains and reached a peak at day 8 p.i.. Resistant strains showed only a slight increase in relative lung weight on day 5 p.i. and 8 p.i.. To confirm that the increase in relative lung weight was not simply due to the loss in body weight at the respective days p.i., we also calculated relative lung weight for the different days p.i. with reference to the starting body weight. Also in this case, we still observed a significant increase in relative lung weight in susceptible strains and no significant increase in the two resistant strains (data not shown).

### Kinetics of viral load in lungs revealed large differences between resistant and susceptible CC founder strains

**Fig. 5:**
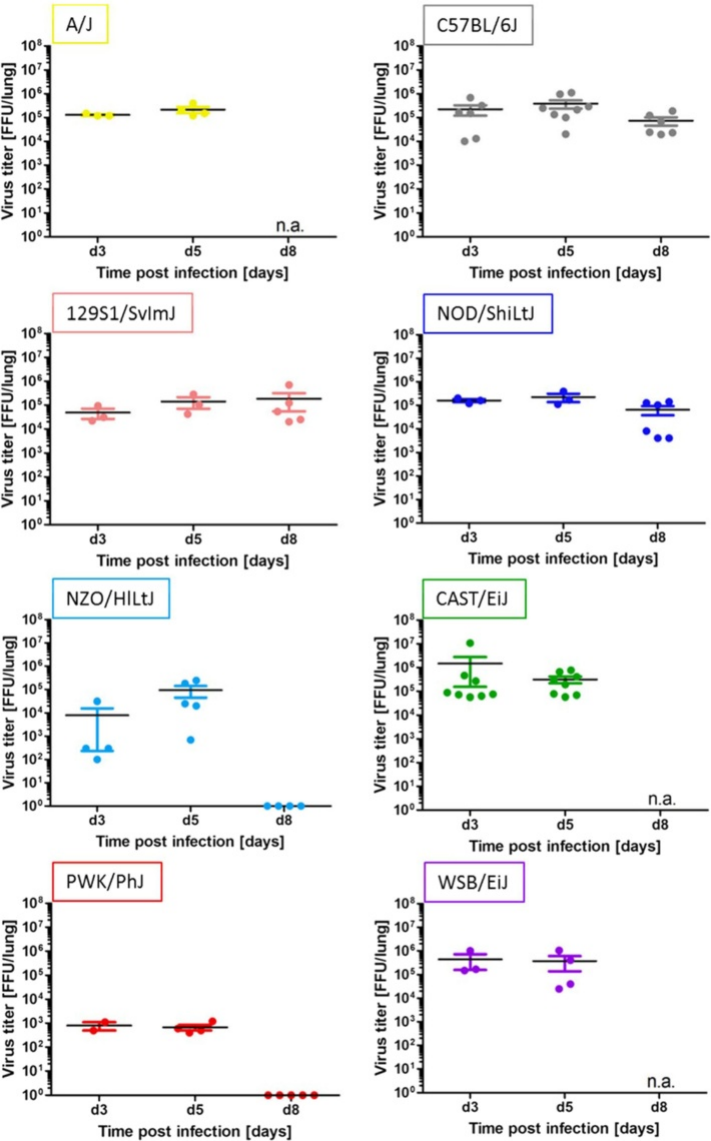
Constant high viral load in highly and intermediate susceptible and viral clearance in resistant strains. Eight to twelve weeks old female mice of the eight CC founder strains were infected intra-nasally with 1 × 10^1^ FFU of influenza H3N2 virus. Lung samples were taken for mock infected animals on day 3 post treatment and on day 3, 5, and 8 p.i.. Lungs of mice of all CC founder strains showed comparable high viral loads at 10^5^ FFU/lung from day 3 until day 5 p.i.. NZO/HlLtJ and PWK/PhJ had lower viral loads and cleared virus until day 8 p.i.. Each data point represents the measurement of one animal; mean and standard error of the mean are shown for each time point and strain

To investigate if the severity of infection was related to viral replication in the lungs of mice, we determined viral load at different time points (day 3, 5 and 8) post infection. All highly and intermediate susceptible strains (A/J, CAST/EiJ, WSB/EiJ, C57BL/6J, 129S1/SvlmJ, NOD/ShiLtJ) showed similar high viral loads of about 10^5^ FFU at all time points analyzed (Fig. 5). In contrast, the highly resistant strains NZO/HlLtJ and PWK/PhJ had much lower viral titers in the lung (Fig. 5). PWK/PhJ exhibited lowest viral loads of all strains (10^3^ FFU/lung) and no increase between day 3 and 5 whereas NZO/HlLtJ mice showed an increase in viral load from day 3 to day 5 p.i. (Fig. 5). Both strains had cleared the virus from their lungs by day 8 p.i.. Viral load was significantly different on day 3 and day 5 p.i. between susceptible and resistant mice but not different between highly and intermediate susceptible mice (ANOVA, model: lg.viral.ld ~ suscept; Additional file 4: Table S4 and Additional file 5: Table S5).

### Each CC founder strain exhibits a characteristic leukocyte profile in peripheral blood

**Fig. 6:**
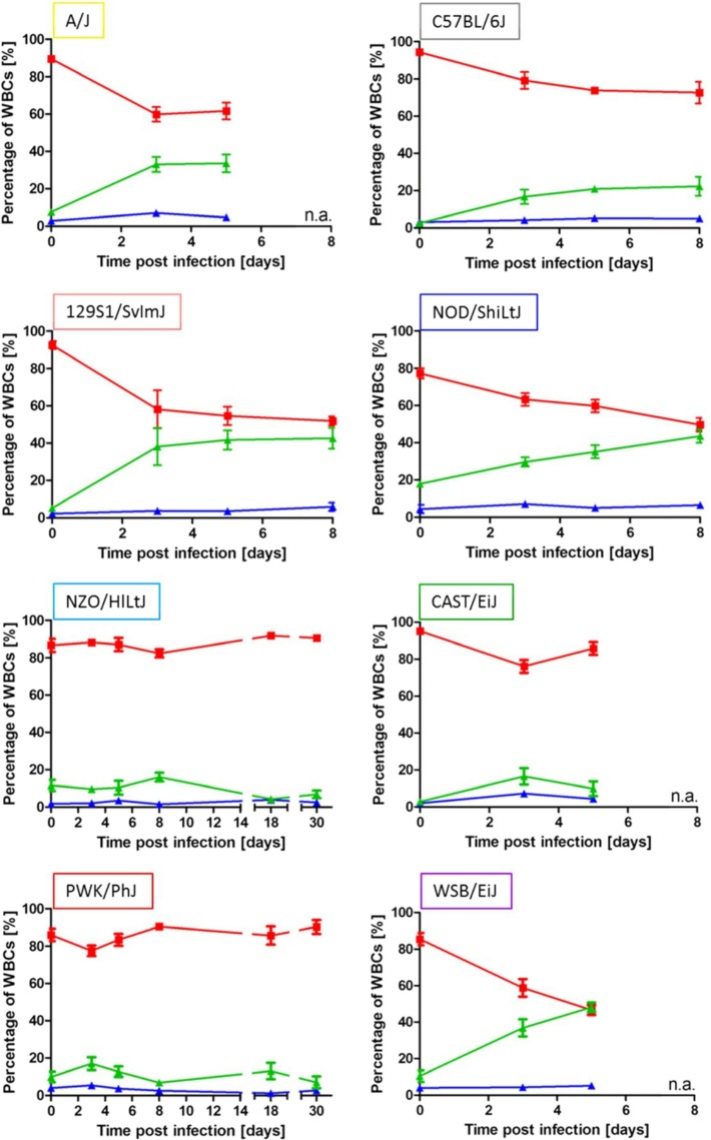
Mice of each founder strain showed a characteristic leukocyte profile in peripheral blood. Blood samples were taken from the same animals used for viral load analysis (Fig. 5) and analyzed regarding granulocyte (green), lymphocyte (red) and monocyte (blue) amounts displayed as percentage of white blood cells (WBCs)

The changes in cellular composition of the peripheral blood after infection was monitored via VetScan hematological assay (Fig. 6). After infection, the percentage of lymphocytes in all strains decreased, whereby the percentage of granulocytes increased. Only the highly resistant strains NZO/HlLtJ and PWK/PhJ showed nearly constant levels of both cell types. The percentage of monocytes was similar for all strains. Comparison between the eight founder strains revealed a high percentage of granulocytes in A/J, 129S1/SvImJ, NOD/ShiLtJ and WSB/EiJ on day 3 and 5 after infection, resulting in a ratio of about 35 % of the leukocytes. C57BL/6J, NZO/HlLtJ, CAST/EiJ and PWK/PhJ exhibited low levels of granulocytes at day 3, 5 and 8 after infection (20 % or below). CAST/EiJ was the only susceptible strain showing a decrease from day 3 to day 5. Notably, granulocyte amounts in mock infected NOD/ShiLtJ mice were at 18 % of total WBCs in mock infection. In conclusion, CAST/EiJ, although carrying high viral loads in the lung, exhibited only very low granulocyte counts in the periphery.

### Correlation analysis of phenotypic traits

**Fig. 7:**
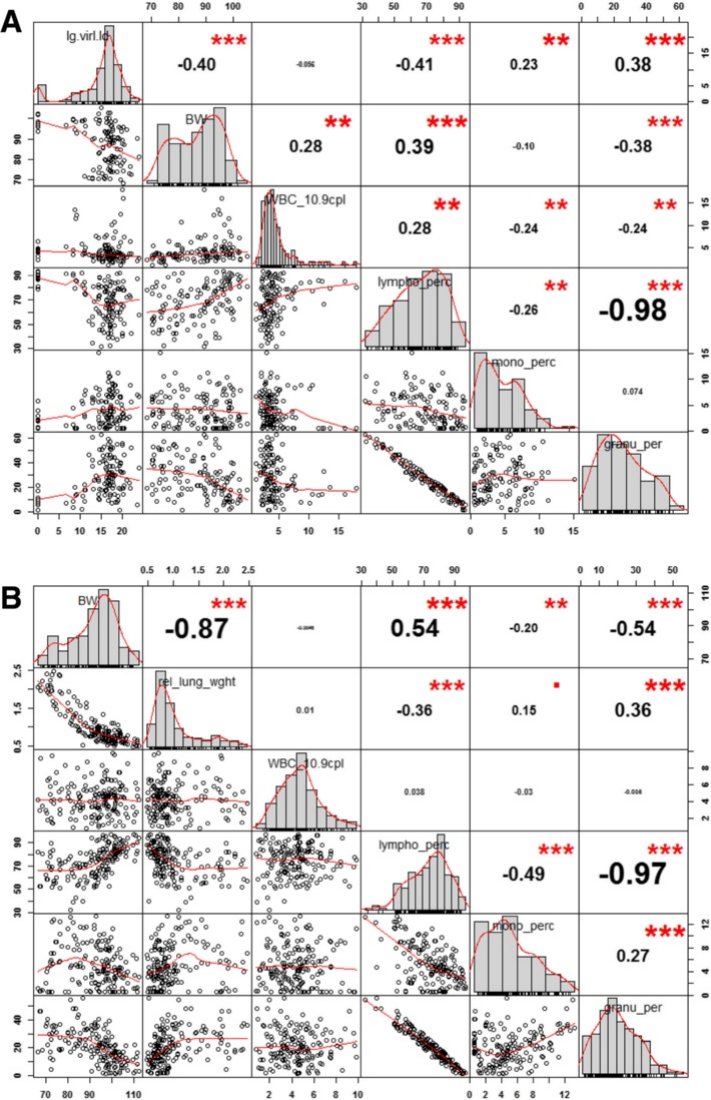
Correlation analysis of phenotypic traits. Correlation analysis of phenotypic traits from setup 2 (**a**) and setup 3 (**b**). The diagonal represents the histogram of the measured values from a given trait, the bottom left panel represents scatter plots of pairs of phenotypic measurements, the top right panel shows correlation coefficients and p-values from a linear regression analysis. Values for days 3, 5, 8 and 18 p.i. (n = 41, 47, 38, and 1, respectively) were combined. BW: percent body weight, re_lung_wght: relative lung weight (in reference to body weight at time of sacrifice), WBC: weight blood cells (10^9^ cells per l), lympho_perc: percent lymphocytes (of total white blood cells), mono_perc: percent monocytes (of total white blood cells), granu_perc: percent granulocytes (of total white blood cells), lg.virl.ld: log_2_ viral load per lung. n = 127 for subset 2, n = 173 for subset 3. **: *p*-value <0.01, ***: *p*-value < 0.001

In our study, we measured changes of several phenotypic traits (body weight, hematology, relative lung weight, viral load) in single mice after infection. This allowed us to correlate these different traits within each subset. Fig. 7a shows the correlation analysis of body weight loss, viral load and percentages of cell populations in the peripheral blood for setup 2 (Table 2). Fig. 7b illustrates the correlation between body weight loss, relative lung weight and percentages of cell populations in the peripheral blood for setup 3 (Table 2). The analysis showed a strong negative correlation (coefficients of –0.98 and –0.97) between percentages of lymphocytes and granulocytes for both subsets which suggests that the main change in peripheral blood cell composition was mainly due to an increase in granulocytes (Fig. 7a, b). Furthermore, we found a strong negative correlation (coefficient of −0.87) between body weight loss and relative lung weight which suggests that the loss in body weight may serve as a surrogate for pathological changes in the lung (edema and immune cell infiltrates) (Fig. 7b). Body weight loss and percentage of lymphocytes appeared only weakly correlated with viral load (Fig. 7a, b). Similarly, body weight loss and percentage of lymphocytes were only moderately or weekly correlated (Fig. 7a, b).

**Table 2:** Experimental setups

Setup	Body weight	Viral load	Hematology	Relative lung weight
1	+	-	-	-
2	+	+	+	-
3	+		+	+

Three different experimental setups with combination of different phenotyping parameters were used

### Detailed analysis of unique CAST/EiJ phenotype by flow cytometry

CAST/EiJ mice were highly susceptible to H3N2 infection and showed a unique combination of high viral load in the lungs (Fig. 5) but low granulocyte counts in the periphery (Fig. 6). Therefore, a more detailed investigation of the CAST/EiJ mice in comparison to two intermediate susceptible mouse strains was performed by flow cytometry of blood and lung cells at day 3 and 5 p.i.. We chose C57BL/6J and 129S1/SvImJ mice for this comparison because 129S1/SvImJ mice exhibited a very strong response whereas C57BL/6J showed an intermediate peripheral response.

**Fig. 8:**
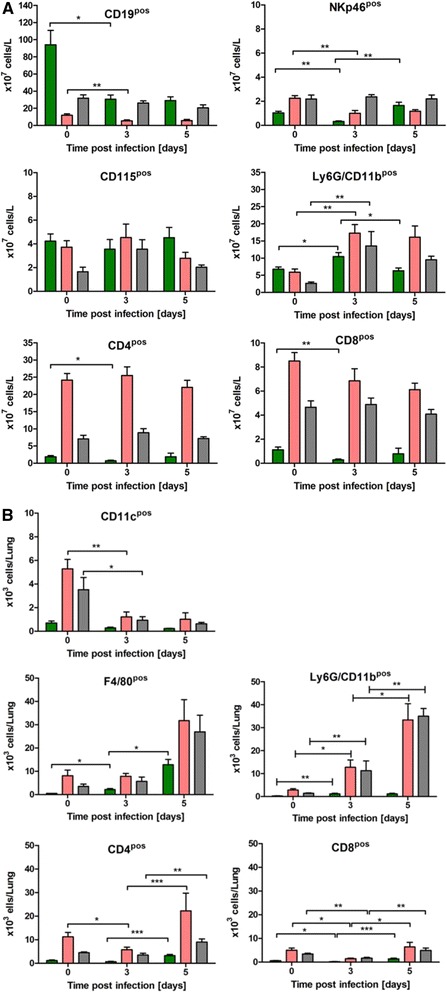
Flow cytometry analysis of immune cells in blood and lungs. Blood samples and lungs from infected C57BL/6J (grey bars), CAST/EiJ (green bars) and 129S1/SvImJ (pink bars) mice (1 × 10^1^ FFU) were prepared. Cell suspensions were analyzed by flow cytometry on days 0, 3 and 5 p.i.. After excluding dead cells fluorochrome-labeled antibodies were used to differentiate various immune cell populations in blood (**a**) and lungs (**b**): CD4^pos^ (T helper cells); CD8^pos^ (cytotoxic cells); CD19^pos^ (B cells), NKp46^pos^ (NK cells), CD11b ^pos^ Ly6G^pos^ (granulocytes), CD115^pos^ (monocytes) and F4/80^pos^ (macrophages). The number of individual cell populations over time (mean +/−SEM) was determined. For each measurement lungs and blood samples from individual animals were used. Data from two independent experiments were combined (n = 6 per time point). Data of different time points were analyzed for statistically significant differences using non-parametric Mann–Whitney-*U*-test (*: *p*-value ≤ 0.05; **: *p*-value ≤ 0.01)

In blood, all three strains had comparable numbers of granulocytes (Ly6G^+^CD11b^+^ cells) before infection (Fig. 8a). At day 3 p.i., the absolute cell numbers of granulocytes increased in all strains, but much less in CAST/EiJ (1.5-fold) compared to C57BL/6J and 129S1/SvImJ mice (5-and 3-fold, respectively) (Fig. 8a). Furthermore, granulocyte counts significantly decreased from day 3 to day 5 in CAST/EiJ (Fig. 8a) but were similar to day 3 in the other two strains (Fig. 8a). After infection, monocyte (CD115^+^ cells) numbers remained high in CAST/EiJ mice at day 5 p.i., whereas they decreased in the other two strains from day 3 to day 5 p.i. (although not statistically significant) (Fig. 8a). Furthermore, NK cell (NKP46^+^ cells) numbers were lower in non-infected and at day 3 p.i. in CAST/EiJ compared to C57BL/6J and 129S1/SvImJ mice and at similar levels in CAST/EiJ mice compared to the other two strains on day 5 p.i. (Fig. 8a). Also, CAST/EiJ mice exhibited the highest absolute numbers of B cells (CD19^+^) in non-infected animals which strongly decreased after infection (Fig. 8a). CAST/EiJ mice also had low numbers of T-helper (CD4^+^ cells) and cytotoxic cells (CD8^+^ cells) compared to C57BL/6 J and 129S1/SvImJ before and after infection (Fig. 8a). Both T-cell populations diminished significantly in CAST/EiJ mice on day 3. In summary, the numbers of granulocytes in the blood were lower after infection in CAST/EiJ mice compared to the other two strains whereas the number of monocytes stayed high in the blood of CAST/EiJ mice.

The most remarkable difference that we observed in flow cytometry analysis of the lungs from infected CAST/EiJ mice (Fig. 8b) was the very low number of all investigated immune cell populations compared to C57BL/6 J and 129S1/SvImJ mice: granulocytes (Ly6G^+^ cells), dendritic cells (CD11c^+^cells), T cells (CD4^+^ and CD8^+^ cells), and macrophages (F4/80^+^ cells). Granulocytes in CAST/EiJ mice increased by 4-fold from day 0 to 3 p.i. with no further increase from day 3 to 5 p.i.. In contrast, granulocyte numbers in the lungs of 129S1/SvImJ and C57BL/6 J increased considerably from day 3 to day 5 p.i. and were 30-times higher on day 5 p.i. compared to CAST/EiJ. The number of macrophages was generally higher in 129S1/SvImJ and C57BL/6 J lungs compared to CAST/EiJ mice. However, the increase in macrophage numbers was much higher in CAST/EiJ (25-fold from day 0 to day 5) in comparison to 129S1/SvImJ and C57BL/6 J lungs (4-and 8-fold, respectively). The amount of dendritic cells (CD11c^+^) decreased similarly in all three strains after infection. CD4^+^ (T helper) cells as well as CD8^+^ (cytotoxic T) cells decreased until day 3 p.i. and then significantly increased until day 5 p.i. in all three strains. In summary, CAST/EiJ mice exhibited in general much lower numbers of immune cells in the lungs before and after infection. After infection, granulocyte numbers increased much less in CAST/EiJ lungs compared to the other two strains whereas the relative increase in macrophages was higher in CAST/EiJ.

### Histopathological and immunohistochemical studies of lungs revealed differences in lung pathology of CAST/EiJ mice in comparison to C57BL/6J and 129S1/SvImJ

**Fig. 9:**
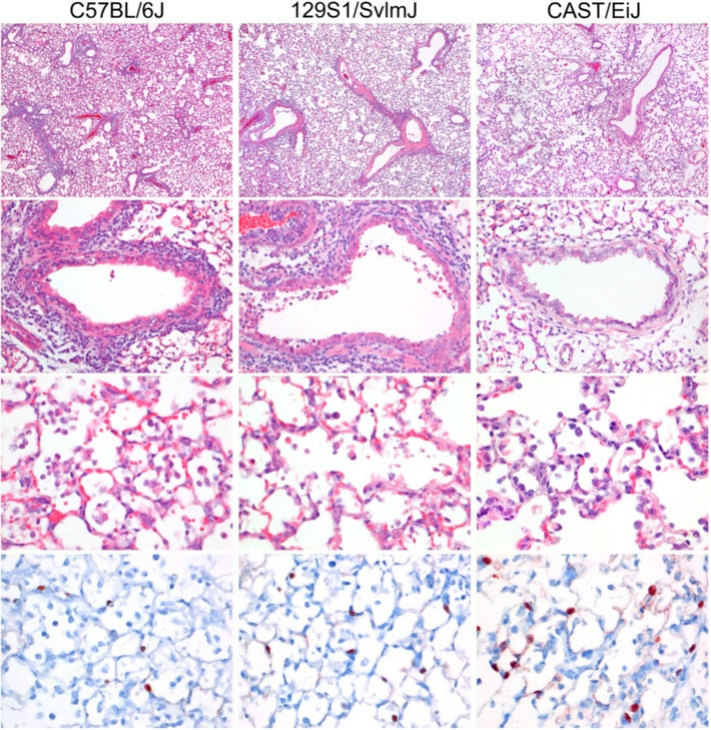
Histopathology and immunohistochemistry of C57BL/6J, 129S1/SvImJ and CAST/EiJ lungs five days after H3N2 infection. The lesions in the lungs of the C57BL/6J and 129S1/SvImJ mice were more extensive than in the CAST/EiJ mice (top row, HE 4× objective) with more severe bronchiolar epithelial necrosis and peribronchiolar inflammatory infiltrates (second row, HE 20× objective), more severe inflammatory infiltrates in the alveoli (third row, HE 40× objective) and more cells with presence of virus antigen by immunohistochemistry (bottom row, IHC 40× objective)

We investigated the lungs of CAST/EiJ mice in comparison to C57BL/6J and 129S1/SvImJ mice by histopathology and immunohistochemistry at different days after infection. C57BL/6J, 129S1/SvImJ and CAST/EiJ mice developed a multifocal mild to severe necrotizing and/or granulocytic broncho-interstitial pneumonia (Fig. 9, Table 3). In C57BL/6J mice at 3 day p.i., the alveolar septa were mildly thickened with infiltration of few granulocytes and there was mild to moderate necrosis of alveolar, bronchiolar and bronchial epithelial cells. In the alveolar, bronchiolar and bronchial lumina there were moderate inflammatory infiltrates consisting of granulocytes, increased numbers of alveolar macrophages, small amounts of proteinaceous material (edema) and fibrin, and mild hemorrhage (Table 3). Peribronchiolarly, peribronchially and perivascularly there was moderate edema and infiltration of moderate numbers of granulocytes and fewer macrophages, lymphocytes and plasma cells. At day 5 p.i., there was an increase in numbers of inflammatory cells in the alveolar lumina and amounts of intraalveolar edema and hemorrhage. Also, there was more alveolar and bronchiolar epithelial necrosis and pneumocyte type II hyperplasia. At day 8 p.i., in addition to previously described changes, there were intra-alveolar hyaline membranes and flattening of bronchiolar and bronchial epithelium. In C57BL/6J mock infected mice no significant lesions were observed. Lungs of 129S1/SvImJ mice showed less granulocyte infiltration and type II pneumocyte hyperplasia in the alveoli, less peribronchiolar, peribronchial, and perivascular infiltration, but slightly more necrosis of alveolar, bronchiolar and bronchial epithelium (Table 3). In lungs of CAST/EiJ mice we observed lower numbers of granulocytes and mild or no peribronchiolar, peribronchial and perivascular infiltrates. There was a trend for fewer intra-alveolar granulocytes and more intra-alveolar macrophages in CAST/EiJ mice compared to C57BL/6J and 129S1/SvImJ mice at day 5 p.i. (Additional file 6: Table S6). These observations were consistent with the flow cytometry data (Fig. 8) showing that CAST/EiJ mice exhibited an increase in granulocytes, macrophages and T lymphocytes whereas the absolute amount of leukocytes remained very low compared to the other susceptible strains. Immunohistochemistry showed that influenza virus antigen was present in respiratory epithelial cells of all strains (Fig. 9, Table 3). The number of positive alveolar epithelial cells peaked at 5 days p.i. in all strains, with about twice as many positive cells in the CAST/EiJ mice as in C57BL/6J or 129S1/SvImJ mice. The number of positive bronchiolar and bronchial epithelial cells was higher in CAST/EiJ mice (day 3 and 5 p.i.) than in C57BL/6J and 129S1/SvImJ mice. This observation revealed that cellular distribution of virus and number of infected cells was different in CAST/EiJ lungs compared to the other highly susceptible strains although the total viral load was similar (Fig. 5). This can be explained by the fact that CAST/EiJ lungs are smaller in comparison to the other analyzed strains. Thus, similar total amounts of infectious virus (viral load) in the lung of CAST/EiJ mice would result in a higher number of infected cells. In mock infected mice no virus antigen was detected in any tissue examined.

**Table 3:** Average scoring of lesions by histopathology and of virus antigen expression by immunohistochemistry in lung tissue of C57BL/6 J, 129S1/SvImJ and CAST/EiJ mice infected with H3N2

		Histopathology score							IHC score	
Strain	Day post infection or mock control	Extent alveolitis/alveolar damage	Severity of alveolitis	Severity of alveolar edema	Presence of interstitial edema	Presence of type II pneumocyte hyperplasia	Severity of bronchiolitis/bronchitis	Degree of peribronchial/perivascular cuffing	Alveoli	Bronchioles and bronchi
C57BL/6J	mock d3	0	0	0	1.0	0	0	0	0	0
	d3	1.7	2.0	0.3	1.0	0.7	2.7	1.7	7.0	1.0
	d5	2.0	3.0	1.0	1.0	0.3	2.3	2.0	9.7	1.3
	d8	3.0	3.0	1.0	1.0	1.0	2.3	1.7	3.0	1.7
129S1/SvImJ	mock d3	1.3	1.0	0	0	0	0	0	0	0
	d3	1.7	2.0	0.3	0.7	0	1.7	1.3	4	0.3
	d5	1.7	2.3	1.0	1.0	0.3	3.0	2.0	10.7	1.3
	d8	3.0	3.0	1.0	1.0	1.0	2.7	2.0	10.3	2.0
CAST/EiJ	mock d3	0.3	0.3	0	0	0	0	0	0	0
	d3	1.7	1.7	0.7	1.0	0.3	1.0	0.7	12	1.3
	d5	2.7	1.7	1.0	1.0	1.0	1.0	1.0	18.7	1.3

### Analysis of global gene expression changes in lung and blood of CAST/EiJ mice revealed abnormal immune response after infection

**Fig. 10:**
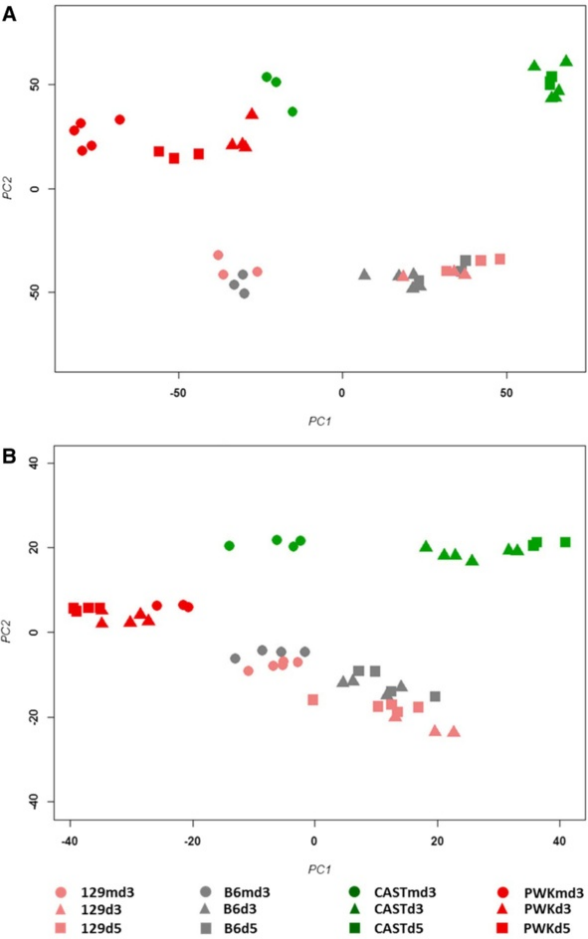
Principle component analysis of gene expression values from infected lungs and blood. Principle component analysis (PCA) revealed separate grouping of non-infected mice and infected mice for all mouse strains in the first component as well as separation by mouse subspecies in the second component. Replicates for a given day p.i. grouped well together. (**a**) PCA of all probe sets from lungs; (**b**) PCA of 2079 probe sets from blood that were significantly regulated (*p* < 0.001). Proportion of variances for lung: PC1: 29 %, PC2: 21 %, PC3: 13 %. Proportion of variances for blood: PC1: 37 %, PC2: 16 %, PC3: 11 %

**Fig. 11:**
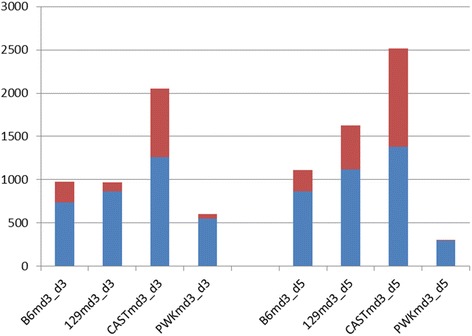
Differentially expressed probe sets. The number of differentially expressed probe sets (DEPS) exhibiting a log_2_-fold change of >1 and an adjusted *p*-value of < 0.05 were determined by LIMMA between infected and non-infected groups for days 3 and 5 p.i.. Up-regulated DEPS are shown in blue, down-regulated in red

To further investigate the high susceptibility of CAST/EiJ mice and to gain insights into the gene regulatory networks involved, we infected female CAST/EiJ, C57BL/6J, 129S1/SvlmJ and PWK/PhJ mice with 1 × 10^1^ FFU H3N2 virus and performed transcriptome analysis of the lungs and peripheral blood at 3 and 5 days p.i. and mock day 3. Principal component analysis (PCA) of all individual samples from the lungs revealed good grouping of biological replicates and a clear separation of infected versus mock-infected mice (Fig. 10a). The first principal component reflected expression changes associated with disease whereas the second principal component was driven by mouse subspecies. C57BL/6J and 129S1/SvlmJ exhibited a change in gene expression from mock-infected to infected lungs at day 3 to day 5 p.i.. Also, CAST/EiJ infected mice did show a strong change from mock-to influenza infected samples. However, samples from days 3 and 5 p.i. were not separated from each other in CAST/EiJ mice in contrast to the changes in C57BL/6J and 129S1/SvlmJ. These observations indicate a strong peak inflammatory response already at day 3 p.i. in infected CAST/EiJ mice. PWK/PhJ mice exhibited a shift from mock-infected mice to day 3 and day 5 p.i. whereby the distance to mock-treated mice was larger at day 3 than at day 5 suggesting that the low replication rate of influenza virus in the lungs of PWK/PhJ mice induced a much lower inflammatory response which was already partly resolved at day 5 p.i.. Identification of differentially expressed probe sets (DEPS, showing a 2-fold or more absolute difference in expression compared to mock-treated mice with an adjusted *p*-value of < 0.05) revealed highest number of DEPS for infected versus mock-infected CAST/EiJ mice on day 3 (2054) and day 5 p.i. (2513), respectively (Fig. 11). C57BL/6J infected mice had intermediate numbers of DEPS that were at similar levels on day 3 p.i. (966 and 975, respectively) and higher in 129S1/SvlmJ (1627) than in C57BL/6J (1108), respectively, at day 5 p.i.. Lowest number of DEPS were observed for PWK/PhJ for both days, exhibiting higher numbers on day 3 (604) compared to day 5 p.i. (306). These results corroborate the conclusions above from the PCA analysis. Analysis of 13 inflammatory genes that are strongly regulated in influenza infected mice [35] revealed that all infected mice exhibited a similar level of up-regulation confirming that all mice were successfully infected. In addition, these results showed that CAST/EiJ mice were able to initiate a proper inflammatory host response (Additional file 7: Figure S1).

For the PCA of the blood transcriptomes, we first performed an ANOVA analysis and selected probe sets that were significantly regulated (*p* < 0.001). The resulting PCA of these 2079 probe sets revealed good grouping of infected and non-infected groups as well as for the different strains (Fig. 10b). Analysis of 13 inflammatory genes [35] showed similar up-regulation of the inflammatory response in CAST/EiJ mice compared to 129S1/SvlmJ and C57BL/6J. PWK/PhJ mice exhibited only weak up-regulation or even down-regulation of these genes (Additional file 8: Figure S2). The results again show that CAST/EiJ mice were capable to initiate a proper inflammatory response at the transcriptional level in the blood.

**Fig. 12:**
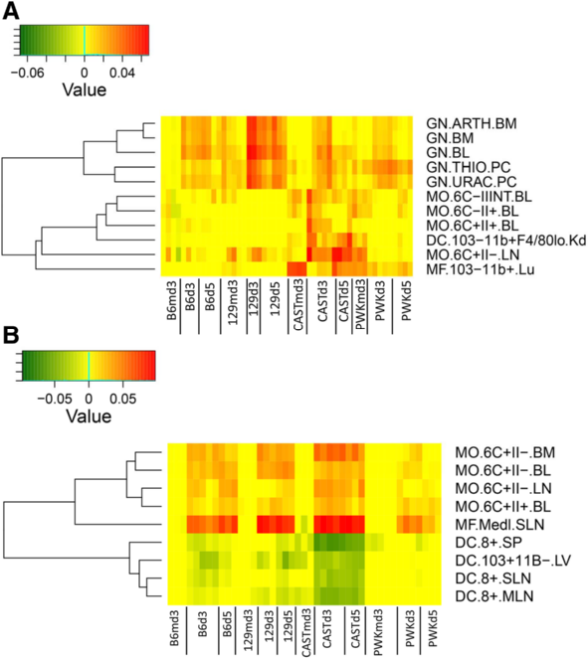
DCQ analysis of gene expression changes in blood and lung. Changes in relative numbers of immune cells were determined by DCQ for each mouse in blood (**a**) and in lung (**b**) based on changes in gene expression levels. Results from the analysis were visualized as heat maps for 217 immune cell populations that are available in the database at the DCQ server (http://dcq.tau.ac.il/). Changes in cell populations that were specific for CAST/EiJ mice are shown. The detailed description of individual cell populations is provided in Additional file 11: Table S7

We also performed a Digital Cell Quantification (DCQ, [36]) to investigate possible differences in immune cell populations of CAST/EiJ mice. DCQ of blood from infected CAST/EiJ mice revealed lower numbers of granulocytes and a higher number of monocytes and macrophages compared to infected 129S1/SvlmJ mice (Fig. 12a). DCQ analysis of the lung transcriptome showed equal numbers of monocytes and macrophages in CAST/EiJ and 129S1/SvlmJ mice and a much stronger reduction in dendritic cells in CAST/EiJ compared to 129S1/SvlmJ mice after infection (Fig. 12b).

**Fig. 13:**
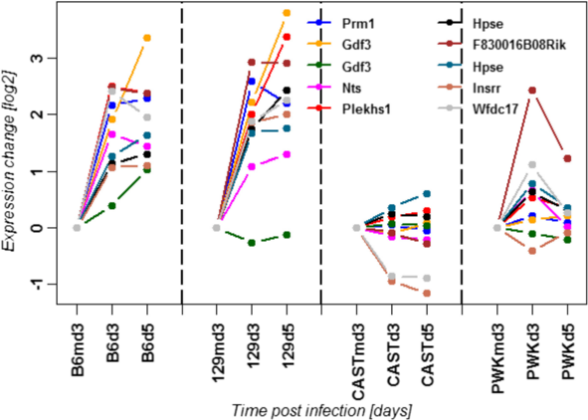
Changes in the expression levels of probe sets that were up-regulated in 129S1/SvImJ and C56BL/6J but not in CAST/EiJ mice. Expression values represent normalized log_2_ transformed signal intensities in the lungs of infected mice at different time points p.i. relative to expression levels in mock-infected control mice

**Fig. 14:**
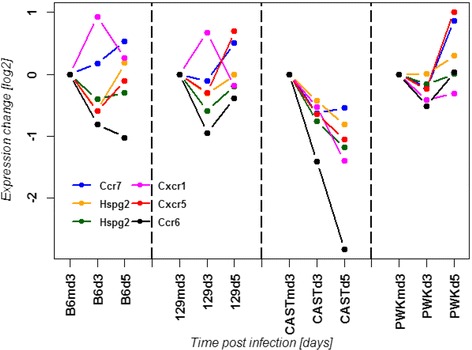
Changes in the expression levels of probe sets that corresponded to genes of the CCR6 regulation network. Expression values represent normalized log_2_ transformed signal intensities in the lungs of infected mice at different time points p.i. relative to expression levels in mock-infected control mice

To identify genes that may be involved in the high susceptibility in CAST/EiJ mice, we determined probe sets that were up-regulated in the lungs of C57BL/6J and 129S1/SvlmJ at days 3 and 5 p.i. compared to the mock control but not in CAST/EiJ. However, it should be noted that differences in expression signals from array analysis of the genetically diverse CC mice may also be caused by SNPs in probes. We therefore confirmed expression changes obtained from an array data set with expression changes observed by RNAseq [31]. Only those genes were retained that were differentially regulated in both, our arrays and the RNAseq study. The filtering revealed eight genes that failed to be up-regulated in CAST/EiJ mice (Fig. 13): *Prm1* (protamine 1), *Gdf3* (growth differentiation factor 3), *Nts* (neurotensin), *Plekhs1* (pleckstrin homology domain containing, family S member 1), *Hpse* (heparanase), *F830016B08Rik* (RIKEN cDNA F830016B08 gene), *Insrr* (insulin receptor-related receptor), *Wfdc17* (WAP four-disulfide core domain 17). In addition, the analysis of the chemokine/cytokine KEGG pathway showed a strong down-regulation of *Ccr6* (chemokine (C-C motif) receptor 6) specifically in CAST/EiJ mice (Fig. 14).

We subsequently investigated other genes involved in the regulatory networks of *Hpse* and *Ccr6* (see discussion) and found specific gene expression changes of *Ccr7* (chemokine (C-C motif) receptor 7), *Cxcr5* (chemokine (C-X-C motif) receptor 5), *Cxcr1* (chemokine (C-X-C motif) receptor 1), and *Hspg2* (perlecan, heparan sulfate proteoglycan 2) in CAST/EiJ mice after infection (Fig. 14).

We also filtered for genes that were up-regulated in the blood of C57BL/6J and 129S1/SvlmJ at days 3 and 5 p.i. but not in CAST/EiJ and found four DEPS: *Il1b* (interleukin 1 beta), *Pi16* (peptidase inhibitor 16) and *I830012O16Rik* (RIKEN cDNA I830012O16 gene). The fourth probe set belonged to a gene from which a second probe set was not lower in CAST/EiJ mice and it was therefore omitted (Additional file 9: Figure S3).

## Discussion

Here, we have analyzed various phenotypic traits after influence A (H3N2) infection in the CC founder strains. We found a strong influence of genetic variation on all traits and a large variation of the host response and pathology. In particular, the response in CAST/EiJ mice revealed a unique phenotype which may represent a new model for the human leucocyte recruitment deficiency disease.

### Susceptibility and resistance to influenza infection and disease is strongly influenced by genetic variation

**Fig. 15:**
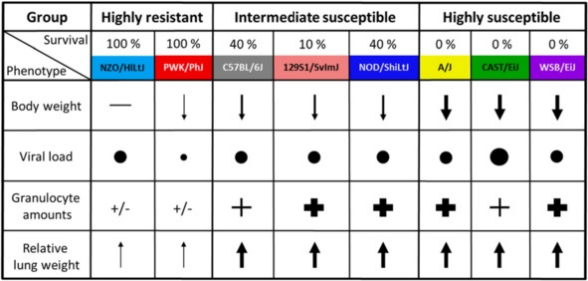
Phenotyping the CC founder strains after influenza A H3N2 infection. The eight CC founder strains were classified into three different subgroups (highly resistant, intermediate susceptible and highly susceptible) according to their outcome after 1 × 10^1^ FFU influenza A H3N2 infection. They showed high variation in changes in body weight, viral loads in lungs, hematological parameters in peripheral blood and relative lung weight

Our comprehensive analysis of the CC founder strains revealed highly variable phenotypes for all investigated traits (Fig. 15). On the basis of mortality rate, MTTD, and body weight changes, we could distinguish three different susceptibility groups: highly susceptible strains, intermediate susceptible strains, and highly resistant strains. Amongst the resistant strains, NZO/HlLtJ only marginally lost weight (less than 10 %) at the low infection dose but exhibited weight loss at the higher doses whereas PWK/PhJ mice also exhibited a larger loss of body weight early after infection.

In previous studies, CC founders and incipient lines of the CC had been studied after infection with H1N1 (A/PR/8/34 (PR8)) virus and the authors described a large variation on phenotypes [34, 37]. Here, we investigated the CC founder strains after infection with another contemporary influenza virus subtype, H3N2 (A/HK/01/68), and we also observed high diversity of phenotypic responses between the founder strains. The study by Ferris et al., phenotyped infected mice only until day 4 p.i. [34]. They identified a highly susceptible and a highly resistant group. Similarly, Bottomly and colleagues categorize a severe and mild response to infection based on global transcriptome profiling [37]. In our studies, we monitored the infected mice over a much larger period, 14 days p.i., and also performed infections with different doses of the same virus, and we distinguish three susceptibility groups. Analysis of variance (ANOVA) for body weight changes revealed that the factor strain had the strongest effect on body weight loss indicating high heritability for this trait. These results thus demonstrate that CC founder strains and CC strains will represent a highly valuable model system to identify genetic factors that regulate susceptibility to infections and other host responses. Indeed, in an analysis of incipient pre-CC strains, several chromosomal regions were identified that influence gene expression of the host response as well as pathology in the lung [34, 37].

### Influence of sex on disease progression and outcome

Using ANOVA, we also found that sex had also a significant effect on body weight loss. At a dose of 1 × 10^1^ FFU female mice exhibited higher body weight loss compared to males. However, the effect of sex was weaker than the strain effect. The influence of sex had not been reported in previous studies which were performed only in females [34, 37]. In pairwise comparisons of strains, we found significant sex-specific differences in body weight loss in the highly and intermediate susceptible group after infection with 1 × 10^1^ FFU, but not in the highly resistant group. Differential responses to H3N2 influenza A infections between female and male mice have been reported previously [38, 39]. Also sex-specific differences to influenza A virus infections in humans have been observed. However, the present knowledge is rather limited because epidemiological data often lack information on sex. Furthermore, tendencies showing that either females or males are more prone to severe disease progression are influenced by additional factors like general life style, social behavior, coinfections or viral exposure (reviewed in: [40]). Our results suggest that influenza infections in the CC population may represent a highly valuable resource to obtain a better understanding of sex-specific responses to pathogens in mammals, including humans.

We did not observe significant differences in survival between male and female mice in the founder stains, except for NOD/ShiLtJ. NOD/ShiLtJ mice spontaneously develop insulin-dependent type 1 diabetes mellitus (T1D) which is characterized by the destruction of pancreatic cells [41, 42]. They also show several abnormalities in immune cells, like defective antigen presentation [43], abnormalities in T [44] and NK cells [45] as well as impaired cytokine signaling in macrophages [46]. Female NOD/ShiLtJ mice exhibit a strong decrease in insulin production already at the age of 12 weeks, which starts several weeks later in males. The incidence of spontaneous T1D is much higher in females (60-80 %) compared to males (20-30 %) [41]. Thus, the increased susceptibility to influenza A infections in females which we observed may be directly related to their pre-diabetic/diabetic state or to an abnormal immune response. It has been shown, that there was an increased risk of complications among patients with diabetes after pH1N1 infection [47]. Therefore, NOD/ShiLtJ mice may provide a valuable mouse model for investigating the role of diabetic state on influenza A infections.

### Different *Mx1* alleles influence survival rates and viral load but are modulated by host genetic background

**Fig. 16:**
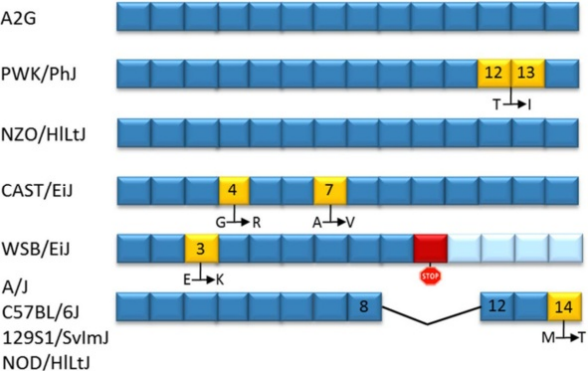
*Mx1* sequence alignment of A2G and the eight CC founder strains. *Mx1* alleles of CC founder strains in comparison to the A2J allele are shown. The mouse *Mx1* gene has 14 exons (blue). Changes of the nucleotide sequence in exons (yellow) result in amino acid exchanges indicated below by showing the respective amino acid exchange. PWK/PhJ exhibits one amino acid exchange relative to A2J, CAST/EiJ two changes at different positions. A premature stop codon is present in WSB/EiJ leading to a truncated MX1 protein. The other strains (A/J, C56BL/6J, 129S1/SvImJ and NOD/ShiLtJ) possess a deletion between the 8th and the 12th exon resulting in a non-functional protein. The graph was created based on data from ncbi.com and [34]

Many studies on influenza pathogenesis in mice were performed in classical laboratory strains which carry a mutation in the influenza resistance gene *Mx1* first described in the A2G mouse strain [48]. The CC founder strains carry five different *Mx1* alleles ([34], Fig. 16). The amino acid sequences of the protein encoded by the *Mx1* alleles in NZO/HlLtJ and A2G were identical to the consensus sequence from the multiple alignments. The PWK/PhJ allele encodes one and the CAST/EiJ allele two amino acid changes compared to the consensus sequence. The WSB/EiJ transcript of *Mx1* harbors a stop codon in its open reading frame leading to a truncated protein. All classical laboratory strains (A/J, C57BL/6J, 129S1/SvImJ and NOD/ShiLtJ) carry a deletion from the 8th to the 12th exon, resulting in a protein that has previously been shown to be non-functional [34, 49]. In our study, the presence or absence of a functional *Mx1* allele correlated strongly with resistance and susceptibility. PWK/PhJ and NZO/HlLtJ were highly resistant and survived a virus challenge with increasing doses of infection, 1 × 10^1^ FFU, 2 × 10^3^ FFU and 2 × 10^5^ FFU. Thus, the single amino acid difference had no influence on the protective functions of the MX1 protein. In contrast, the amino acid exchanges in the CAST/EiJ protein seemed to be detrimental leading to a highly susceptible phenotype. Similar results were obtained after infection with H1N1 (A/PR/8/34 (PR8)) virus [34]. We recently found that the protective function of MX1 is dependent on genetic back ground [50]. Thus, further experiments are needed to elucidate the function of the CAST/EiJ MX1 protein and the potential contribution of the CAST/EiJ genetic background.

MX1 is known to inhibit IAV replication [51, 52]. Therefore, we investigated viral load at different time points p.i. in the CC founder stains and related to the absence or presence of a functional *Mx1* allele. Viral load was lowest in PWK/PhJ mice, was only detected at early times p.i. and was cleared by day 8 p.i.. NZO/HlLtJ mice were also able to clear the virus, but viral titer increased until day 5. Thus, there is a clear difference in viral load even in strains that carry a functional *Mx1* allele which may be caused by genes that modulate the activity of MX1 or other immune response genes. In contrast, all intermediate and highly susceptible mice strains that carried the non-functional *Mx1* alleles exhibited high viral loads already on day 3, and virus was not cleared on day 8 or at the time of death. The CC population now offers the possibility of investigating the same *Mx1* allele on different genetic backgrounds and to identify potential modifying loci that interact with MX1.

### Correlation of viral load with other phenotypes

We did not observe a general correlation between viral load and survival in the susceptible strains. All susceptible and intermediate susceptible strains had similar viral loads. For other mouse strains it had been shown that high viral loads in the lung correlate well with increased susceptibility to IAV. For example, the highly susceptible DBA/2 J strain exhibited a higher viral load compared to resistant C57BL/6J mice [53, 54]. Boon et al. [55] studied viral load in 21 inbred mouse laboratory strains infected with H5N1 (A/HK/213/03). They found significant association of viral load and mortality whereby an increased host inflammatory response was caused by a higher viral load. However, other studies demonstrate no differences in viral load between resistant and susceptible strains [56]. Ferris et al. concluded from studies of pre-CC mice that both viral replication and virus-induced inflammation were predictors for disease severity being independent of each other. Nevertheless, when both were combined they were better predictors than either of them alone [34]. Thus, in conclusion, either increased viral load causing extensive tissue damage or a hyper-inflammatory host response may result in a lethal outcome of an influenza infection. Therefore, the analysis of the divergent host responses in CC mice will allow investigating the biological mechanisms and the cellular and molecular networks in detail in a controlled experimental system and thus substantially contribute to better understand individual host factors that are involved in causing severe influenza disease outcomes in humans.

### CAST/EiJ mice exhibit a distinct deficiency in their immune response to influenza infections

Hematological analysis of the peripheral blood in infected CAST/EiJ mice showed only a slight increase in granulocytes in the peripheral blood compared to other strains that had high viral loads in their lungs. Thus, we hypothesized that CAST/EiJ may exhibit a specific deficiency in their response to influenza infection. We therefore studied the immune response in CAST/EiJ mice in more detail by analyzing immune cells in blood and lung in comparison to other strains. Flow cytometry studies revealed that the increase of granulocytes in the blood was much lower after infection in CAST/EiJ mice compared to the other two strains whereas the number of monocytes stayed high in the blood of CAST/EiJ mice. In the lung, CAST/EiJ mice exhibited an increase of granulocytes, macrophages and T lymphocytes. However, the absolute amount of leukocytes remained very low compared to the other susceptible strains. After infection, granulocyte numbers increased much less in CAST/EiJ lungs compared to the other two strains whereas the relative increase in macrophages was higher in CAST/EiJ. Histological analysis confirmed the low level of neutrophil infiltrations in the lung and increased macrophage numbers. Also, DCQ analysis of global gene expression changes confirmed the low neutrophil response in the blood, reduced neutrophil infiltration into the lung and an increase in macrophage numbers in the lung. In line with our results, Ferris et al. showed that CAST/EiJ mice developed high viral loads in lungs after influenza infection but showed less infiltrating immune cells in their lungs [34]. They and others observed low levels of T and NK cells in the blood of CAST/EiJ mice [34, 57]. CAST/EiJ mice are also highly susceptible to monkeypox virus (MPXV) infection lacking interferon gamma (IFN-γ) induction which might be related to a defect in activation and recruitment of IFN-γ producing immune cells [58]. CAST/EiJ mice were rescued from lethal challenge with MPXV by intra-nasal administration of IFNγ [58]. However, IFNγ is not crucial for the host response to influenza infection [59] whereas, interferons type I and III are essential [60–62]. Therefore, we investigated if pre-treatment with IFN-I was able to rescue CAST/EiJ mice from lethal influenza A infection. We observed a shift of about two to three days in the survival curve for IFN-treated versus non-treated CAST/EiJ mice. However, IFN pre-treatment did not rescue CAST/EiJ mice from lethal infection (data not shown). In addition, we did not observe reduced levels of *Ifng* transcripts in CAST/EiJ compared to the other mouse strains (Additional file 7: Figure S1 and Additional file 8: Figure S2).

Our observations suggest that granulocytes have a deficiency to enter the site of infection in CAST/EiJ mice. Members of the CXC chemokine family, notably CCL4 and CCL5, as well as selectins, play a major role in the recruitment of leukocytes, especially of neutrophils [63]. We showed earlier that genes from these two families, notably CXC chemokines, are upregulated very early after influenza infection in lungs of C57BL/6J mice [35]. However, we observed that after infection the transcript levels of *Ccl4* and *Ccl5* were similarly up-regulated in the lungs of CAST/EiJ as for 129S1/SvImJ and C57BL/6J (data not shown). Also, deficiencies in several CXC chemokine ligands and receptors (*Cxcr2*, *Cxcl10*, *Cxcr3*, *Cxcl5*) diminish the leukocytes influx into lungs [64–67]. The expression of *Cxcl10*, *Cxcr3* and *Cxcl5* was not differently regulated in CAST/EiJ compared to the two other susceptible mouse strains (data not shown). However, expression of *Cxcr2* dropped to base line levels at day 5 p.i. only in CAST/EiJ mice (data not shown). It was reported for various inflammation models that in mice deficient in E-, L-, or P-selectin genes (*Sele*, *Sell*, *Selp*), the levels of neutrophils, T lymphocytes and lung inflammation are reduced [68–72]. All selectin genes were similarly up-regulated in all three susceptible genes in the lung after infection. However, *Sele* was strongly down-regulated in CAST/EiJ on day 5 p.i. (Additional file 10: Figure S4). Furthermore, the continuous recruitment of leukocytes is regulated by a feed-forward loop in which infiltrated immune cells in the lung enhance the release of chemokines leading to the recruitment of more neutrophils [73]. The depletion of neutrophils resulted in uncontrolled virus growth and increased mortality in mice [74]. Thus, reduced granulocyte infiltration in CAST/EiJ may secondarily also affect the subsequent recruitment of more granulocytes and other immune cells. However, we do observe an increase in macrophages. Further analyses will be necessary to unravel the details of these interactions phenomenon.

In summary, the amount of granulocytes in the blood of CAST/EiJ mice increased after infection which suggests a normal primary signaling of infected cells to the hematopoietic system and the release of leukocytes into the blood. However, the number of granulocytes remained constant in the lungs of CAST/EiJ from day 3 on and did not further increase. In contrast, granulocyte numbers strongly increased in lungs of 129S1/SvImJ and C57BL/6J mice. The strong specific down-regulation of *Sele* at day 5 supports the hypothesis of a leukocyte recruitment deficiency in CAST/EiJ mice. Together, these results suggest that the lack of sustained neutrophil recruitment may be the primary defect in CAST/EiJ mice.

We also performed a more detailed analysis of gene expression changes at the level of gene interaction pathways and individual genes to obtain more insights into the underlying mechanism of the abnormal CAST/EiJ immune cell phenotype. The canonical inflammatory genes, interferons and interferon response genes as well as many inflammatory chemokines such as *Cxcl10*, are all highly up-regulated in the lungs of CAST/EiJ mice. In addition, CAST/EiJ mice showed the highest number of DEPS after infection in blood and lungs. Thus, CAST/EiJ mice exhibited not a general deficiency in the overall activation of an inflammatory response but rather a specific defect in the recruitment of cells to the lung. Seven genes were specifically not up-regulated in CAST/EiJ after infection which have not been described in the context of the host response to influenza infections, but may be also important host factors for virus defense in humans [14]. Furthermore, the *Hpse* gene was not as strongly up-regulated after infection in CAST/EiJ compared to the other mouse strains. *Hpse* encodes heparanase, a mammalian endoglycosidase which degrades heparan sulfate glycosaminoglycan in extracellular space and within the cells. It was postulated that heparanase may play an important role in inflammatory and autoimmune processes including leukocyte recruitment, immune cell extravasation and migration, release of cytokines and chemokines and activation of innate immune cells (reviewed in [75, 76]). Recently, the role of heparanase in regulating the migration of DCs was demonstrated in heparanase-deficient mice [77]. When we analyzed the expression profiles of genes in the heparanase network, we found a specific down-regulation of the *Hspg2* gene in CAST/EiJ mice. *Hspg2* encodes perlecan [78–80] also known as basement membrane-specific heparan sulfate proteoglycan 2 (HSPG2). Perlecan is a large multi-domain proteoglycan that binds to and cross-links many extracellular matrix (ECM) components and cell-surface molecules. It is a key component of the vascular extracellular matrix, where it interacts with a variety of other matrix components helping to maintain the endothelial barrier function. Three long chains of glycosaminoglycans are attached to the perlecan core protein that plays an important role in the recruitment of leukocytes via binding to chemokines [81, 82]. Also, the *Wfdc17* gene, encoding the AMWAP protein (activated microglia/macrophage whey acid protein domain protein), was not expressed in CAST/EiJ mice before and after infection. AMWAP has been postulated to be a negative regulator of pro-inflammatory microglia/macrophage activation and a potential modulator of innate immunity in neurodegeneration [83]. Interestingly, *Wfdc17* was up-regulated in lungs of C57BL/6J, 129S1/SvlmJ and PWK/PhJ after infection, but not in CAST/EiJ which is consistent with the observed increase of infiltrating macrophages in CAST/EiJ lungs. In addition, analysis of the KEGG chemokine/cytokine pathway revealed a strong down-regulation of *Ccr6* (chemokine (C-C motif) receptor 6) in the lung that was unique for CAST/EiJ mice. CCR6 is an important chemokine receptor that mediates recruitment of immature and mature DCs and antigen presenting cells (APCs) to the sites of epithelia inflammation in the lung (reviewed in [84]). Epithelial cells in the lung increase their production of CCL20, which is the only chemokine ligand for CCR6, which in turn causes a further recruitment of immature CCR6-positive DCs into the lung. *Ccr6* expression is down-regulated during the maturation process and accordingly *Ccr7* is upregulated leading to the migration of DCs to the lymph node to fulfill its APC function. The expression of *Ccr6* was down-regulated in all analyzed mouse strains, but very strongly in CAST/EiJ lungs, especially at day 5 after infection. Interestingly, *Ccr7* expression was up-regulated in all strains, but not in CAST/EiJ, where *Ccr7* was down-regulated. After infection a large number of immature DC continuously enter the lung tissue and then differentiate into mature DCs [84]. This process was reflected by the up-regulation of *Ccr7* in C57BL/6J and 129S1/SvlmJ. However, in CAST/EiJ mice less immature DC cells enter the lung which may be reflected by the decrease of *Ccr6* and *Ccr7* expression. Another down-regulated gene in CAST/EiJ was *Cxcr1*, a member of the G-protein-coupled receptor family. CXCR1 is a receptor for the CXC chemokine interleukin IL-8, which is a potent neutrophil recruiting [85] and activating factor [86, 87]. IL-8 is an important modulator of monocyte-endothelial interactions under flow conditions, since it can rapidly cause rolling monocytes to adhere firmly onto monolayers expressing E-selectin [88]. Furthermore, *Cxcr5* was down-regulated in CAST/EiJ lungs but not in the other strains. CXCR5 is a cytokine receptor that binds to B-lymphocyte attractant (BLC) and is involved in B-cell migration [89, 90].

Filtering for genes that were up-regulated in the blood of C57BL/6J and 129S1/SvlmJ but not in CAST/EiJ identified three genes. One of those *Il1b* (interleukin 1 beta) encodes IL1B which is an important inflammatory cytokine that mediates the inflammation and initiate the immune response against Influenza A virus infection [82]. Pro-IL-1B is cleaved by caspase-1 which is activated through the formation of the NLRP3 inflammasome [91] and induces the expression of a variety of inflammatory mediators, which initiate the cascade of inflammatory responses [92]. It was postulated that the synergy between CD40L and IL-1beta represents a potent, T cell-independent mechanism for DC activation during the earliest stages of inflammatory responses [93]. Most interestingly, in humans it has been described that genetic variants in IL1A and IL1B contribute to the susceptibility to 2009 H1N1 infections [94].

In summary, the specific changes of gene expression patters that were unique in CAST/EiJ lungs belong to gene networks that play a crucial role in migration and recruiting of leukocytes. Together, these observations suggest a deficiency in CAST/EiJ mice in leukocyte adhesion and/or recruitment. The recruitment of leukocytes to the site of infection is a complex process of different steps including leucocyte rolling, activation, arrest and transmigration into the tissue which involves many proteins and interactions. Three types of leukocyte adhesion deficiencies (LADI-III) have been described in humans (reviewed in [95]). Mutations in ITGB2 (integrin beta 2), SLC35C1 (solute carrier family 35) and FERMT3 (fermitin family homolog 3) have been identified as the genetic cause of disease in LADI, II and III, respectively [95]. However, we did not observe differences in the expression of these LAD genes in CAST/EiJ mice (data not shown). Thus the specific CAST/EiJ phenotype may be a valuable model system to identify and study additional genes related to human leukocyte adhesion and recruitment deficiencies. Of course, further functional analyses under different conditions will be necessary to describe the leukocyte phenotype in CAST/EiJ mice in more detail.

## Conclusion

In conclusion, our studies on the CC founder strains emphasize the importance of host genetic backgrounds for the course and outcome of IAV infections. The CC founder strains as well as the CC strains will thus represent an extremely valuable experimental model system to understand the biological mechanisms as well as the genetic factors that cause severe influenza disease in humans. In most cases, high mortality was associated with high viral loads and a strong immune response. However, we also observed unexpected phenotype combinations such as high viral load but a weak immune response. In particular, the CAST/EiJ strain may provide a novel model for human leukocyte adhesion and recruitment deficiencies.

## Methods

### Ethics statement

All experiments in mice were approved by an external committee according to the national guidelines of the animal welfare law in Germany (BGBl. I S. 1206, 1313 and BGBl. I S. 1934). The protocol used in these experiments has been reviewed by an ethics committee (equivalent to US IACUC, according to the German law) and approved by the ‘Niedersächsisches Landesamt für Verbraucherschutz und Lebensmittelsicherheit, Oldenburg, Germany’ (Permit Numbers: 33.9.42502-04-051/09 and 3392 42502-04-13/1234).

### Virus, mouse strains, infection

The mouse-adapted virus strain influenza A/HK/01/68,obtained from Otto Haller, University of Freiburg [96] was produced in the allantoic cavity of 10-day-old embryonated hen eggs for 48 h at 37 °C. The CC founder (A/J, C57BL/6J, 129S1/SvlmJ, NOD/ShiLtJ, NZO/HILtJ, CAST/EiJ, PWK/PhJ, WSB/EiJ) were purchased from Jackson Laboratories (Bar Harbor, ME) and bred in our animal facility in Braunschweig for two to six generations depending on the respective strain. All mice were maintained under specific pathogen free conditions and according to the German animal welfare law. The entire age range of mice was 7 to 13 weeks whereby 95.5 % of the mice had an age range of 8–12 weeks. Female and male mice were anesthetized by intra-peritoneal injection with a mixture of Ketamine/Xylazine (100 mg/ml Ketamine and 20 mg/ml Xylazine) in sterile natrium chloride solution. The doses were adjusted to the individual body weight using 200 μl/20 g body weight. Mice were then intra-nasally infected with 20 μl virus solution of sterile phosphate-buffered saline using different virus concentrations (1 × 10^1^, 2 × 10^3^ and 2 × 10^5^ FFU). Body weight was determined daily as % of initial weight at day 0. Mice that lost more than 30 % of their body weight had to be killed for ethical reasons and were also scored as dead. Three different experimental setups with combination of different phenotyping parameters were used (Table 2).

### Relative lung weight

Mock-infected and infected female mice (n = 3-6 per strain and time point) were anesthetized using 160 μl Isofluoran (cp-pharma, Burgdorf, Germany). After exsanguinating via puncture of the retro-bulbar vein plexus, lungs were collected and weighted directly. Relative lung weight was calculated as followed: (lung weight on day of preparation/body weight on day of preparation) × 100 and displayed as percentage of total body weight.

### Viral load in lungs

Viral load in infected lungs was determined on MDCK II (Madin-Darby Canine Kidney II) cells using the FFU assay as described previously [53]. Briefly, lungs of mice were homogenized and debris removed by centrifugation. Samples were stored in aliquots at −70 °C. Serial dilutions of lung homogenates were prepared and viral titers determined by the FFU assay.

### RNA isolation for arrays

At different time points after virus infection (day 3 and 5) and mock infection (day 3 post sterile phosphate-buffered saline infection) lung and blood were taken for RNA isolation. Body weight was measured until the day of sample collection. Eye blood was collected in RNA protect animal blood tubes (Qiagen) and immediately stored at −20 °C. Lung RNA was isolated using Qiagen Midi Kit as described previously (104). Blood RNA was isolated with the RNeasy protect animal blood kit (Qiagen). RNA concentration was measured with the NanoDrop (Thermo Scientific).

### Microarray experiment

For DNA microarray hybridization and analysis, the quality and integrity of the total RNA was controlled on a 2100 Bioanalyzer (Agilent Technologies; Waldbronn, Germany). 100 ng of total RNA were applied for Cy3-labelling reaction using the one color Quick Amp Labeling protocol (Agilent Technologies; Waldbronn, Germany). Labeled cRNA was hybridized to Agilent’s mouse 4 × 44 k microarrays for 16 h at 68 °C and scanned using the Agilent DNA Microarray Scanner. Expression values were calculated by the software package Feature Extraction 10.5.1.1 (Agilent Technologies; Waldbronn, Germany).

### Statistics

Data were analyzed using GraphPad Prism version 5.04 for Windows (GraphPad Software, San Diego, California). Mean and standard error of the mean (SEM) were calculated for all groups. Dunn’s post test with multiple testing correction was used for significance testing between individual groups (e.g. different gender, virus concentration and time points). Non-parametric Mann Whitney *U* test was used to determine *p*-values for the significance of differences between two groups. Log rank test was used to calculate significances for survival rates. Analysis of variance (ANOVA) was performed using the lm and aov functions of the R software package [97]. A multi-factorial ANOVA model (weight-loss ~ strain * sex * day) with all interaction terms and with days (from day 1 to day 7 p.i.) as a categorical factor was used first. In this analysis, the three-way interaction term strain:day:sex was not significant and was therefore removed to generate an optimized ANOVA model (Additional file 2: Table S2). Subsequent Tukey HSD post-hoc test was used to identify pairwise significant differences. A one-way ANOVA was performed using the model weight-loss ~ strain for each individual day separately, and the broad-sense heritability was determined by calculating the interclass correlation [98]: (MSB-MSW)/(MSB+(n-1)*MSW) with MSB = mean square between groups, MSW = mean square within groups, n = mean number of mice per strain. One way ANOVA (model lg.viral.ld ~ suscept, see (Additional file 4: Table S4 and Additional file 5: Table S5 for details) and Tukey HSD post-hoc test was used to determine differences of viral load between strains of different susceptibility (intermediate susceptible, highly susceptible and resistant strains). Correlations for phenotypic traits were calculated and illustrated using R scripts developed by W. Venables (http://goo.gl/nahmV), S. Turner (https://gist.github.com/stephenturner/3492773) and the graphics from the package PerformanceAnalytics (https://cran.r-project.org/).

### Gene expression analysis

Array data were analyzed using the R software package [97]. Pre-processing steps included background correction, quantile normalization and annotation using the MmAgilentDesign026655.db [99], limma [100], and Agi4 × 44PreProcess [101] packages. Principal component analysis (PCA) analysis were performed using the affycoretools package [102]. Multi-group comparisons and identification of differentially expressed probe sets (DEPS) were performed with the LIMMA package [100] using BH correction for multiple testing [103]. DEPS were identified based on an adjusted p-value of < 0.05 and exhibiting more than a two-fold difference in expression levels (abs [log_2_] > 1). Inflammatory genes that are expressed during influenza infections were selected based on our previous influenza transcriptome studies [35, 104]. GO, KEGG, Reactome enrichment analysis and cluster profiling was performed with the package clusterProfiler [105]. For the identification of genes that exhibited no change in gene expression levels after infection in the lungs of CAST/EiJ mice compared to the other strains, the full expression data set was filtered for probe sets that were not regulated in CAST/EiJ (log-fold change absolute <0.5) but up-regulated in C57BL/6J and 129S1/SvlmJ mice at days 3 and 5 after infection (log-fold change absolute >1). In addition, the data set was filtered for probe sets that were down-regulated in CAST/EiJ (log-fold change < −0.5) but up-regulated in C57BL/6 J and 129S1/SvlmJ mice at days 3 and 5 after infection (log-fold change absolute >1). These filters returned 8 and 2 probe sets, respectively. It should, however, be noted that gene expression differences between strains which are observed in Agilent arrays may not always represent true differences in expression levels between strains but may also result from sequence polymorphisms in transcripts that result in mismatches between probes and transcripts, since the probes on the array are based on the C57BL/6J reference strain. Therefore, we validated true expression changes by analyzing the respective genes in a previously published RNAseq data set [31]. Only genes that were also differentially expressed in the RNAseq data set were further investigated. In this way, eight genes were found to be regulated in CAST/EiJ versus the other strains. The same analysis was performed for blood transcriptomes. Four DEPS were not regulated in CAST/EiJ (log-fold change absolute <0.5) but up-regulated in C57BL/6J and 129S1/SvlmJ mice at days 3 and 5 after infection (log-fold change absolute >1). KEGG pathway analysis was performed using the pathview package in R [106]. DCQ analysis was performed according to the algorithm described in [36]. Gene expression values were pre-processed by subtracting the mean of the mock-infected mice from the infected mice for each mouse strain and by selecting the most variant probe sets for genes with multiple probe sets based on the standard deviation across all groups. DCQ analysis was then performed at the public server provided by the University of Tel Aviv (http://dcq.tau.ac.il/). The results were then visualized by heatmaps using the R software [97].

### Histology

Tissues were stored in 10 % neutral-buffered formalin (lungs after careful inflation with formalin), embedded in paraffin, sectioned at 4 μm, and stained with hematoxylin and eosin (HE) for examination by light microscopy. Semi-quantitative assessment of influenza virus-associated inflammation by histopathology in the lung was performed as reported earlier [107] for the extent of alveolitis and alveolar damage we scored: 0, 0 %; 1, 1-25 %; 2, 25-50 %; 3, > 50 %. For the severity of alveolitis, bronchiolitis and bronchitis we scored: 0, no inflammatory cells; 1, few inflammatory cells; 2, moderate numbers of inflammatory cells; 3, many inflammatory cells. For the presence of alveolar edema, alveolar hemorrhage and type II pneumocyte hyperplasia we scored: 0, no; 1, yes. Finally, for the extent of peribronchial, peribronchiolar and perivascular infiltrates we scored: 0, none; 1, one to two cells thick; 2, three to ten cells thick; 3, more than ten cells thick. Slides were examined without knowledge of the animals.

### Immunohistochemistry

For detection of influenza A virus antigen, tissues were stained with a primary antibody against the influenza A nucleoprotein as described previously [108]. In each staining procedure, an isotype control was included as a negative control. Semi-quantitative assessment of influenza virus antigen expression in the lungs was performed as reported earlier [107] with minor modification: for the alveoli, 25 arbitrarily chosen, 20× objective fields of lung parenchyma per animal were examined by light microscopy for the presence of influenza virus nucleoprotein, without the knowledge of the identity of the animals. The score for each animal was presented as the percentage of positive fields. For the bronchi and bronchioles, the percentage of positively staining epithelium was estimated on every slide to provide the score per animal: 0, 0 %; 1, 1-25 %; 2, 25-50 %; 3, > 50 %.

The numbers of neutrophils and intraluminal macrophages were counted in 20 arbitrarily chosen, 40× objective fields per animal. Slides were examined without knowledge of the identity of the animals. Viable neutrophils were identified by immunohistochemistry staining with primary antibody Ly-6G (rat-anti-mouse Ly-6G, 25–5931, eBioscience, San Diego, USA). Binding of the primary antibody was detected using a peroxidase labeled rabbit-anti-rat IgG-HRP (p0450, DAKO, Denmark). Peroxidase activity was revealed using 3-amino-9-ethylcafrbazole (AEC) (Sigma, St Louis, MO, USA). Intraluminal macrophages were identified on the basis of their morphology and location as described previously [109].

### Hematology

For monitoring hematological parameters, female mice were anesthetized using 160 μl isoflourane at different time points (mock day 3, day 3, 5, 8, 18 and day 30) after infection. Time points were omitted from the analysis when less than 60 % of the infected mice survived infection within a strain. Blood was taken by puncture of the retrobulbar vein plexus, collected in micro tubes containing EDTA (Sarstedt) and immediately measured in the hematologic system VetScan HM5 (Abaxis). The absolute cell numbers of lymphocytes, granulocytes and monocytes were determined and calculated as percentage of white blood cells.

### Flow cytometry

Immune cell populations of single animals were analyzed in cell suspensions derived from homogenized lungs or whole blood. Blood was collected as described for the haematological analysis. Antibody against CD16/32 (clone 2.4G2) was added to blood minimizing unspecific binding to Fcy receptors. Blood cells were directly stained in two different panels with the following antibodies: αCD4-PE (BD Bioscience, Europe), αCD8-APC, αCD11b-APC, αCD19-FITC, αCD115-Alexa Fluor 488, αLy-6G-PE-Cy7 and αNKp46-PerCP-eFluor 710 (eBioscience). After incubation at 4 °C for 30 minutes, in the dark, erythrocytes were lysed and cells fixed in FACS Lysing Solution (BD Bioscience), and washed twice with PBS/2%FCS. Lungs prepared from the same animals were passed through a 100 μm-cell strainer and a density centrifugation using Lympholyte M (Cedarlane) was performed. After washing the cells obtained from the interphase twice with PBS/2%FCS, Fcy receptors were blocked with αCD16/32 and subsequently stained with antibodies as described above. In addition αF4/80-FITC and αCD11c-PE (eBioscience) were used. To exclude dead cells, 1 μg propidium iodide was added to each sample. Samples were measured using an Accuri C6 flow cytometer (BD Bioscience). Data were analyzed using FlowJo version 7.6.5 (Tree Star, Ashland, Oregon). The marker expression and gating strategy of immune cell populations was done as described [53]. Minor subpopulations like CD8 + DC were not considered.

### Deposition of data

Array data have been deposited at the gene expression data base GEO (GSE74077), phenotype data will be deposited at the mouse phenome database (MPD, [110]) upon publication of the manuscript.

## Additional files

Additional file 1: Table S1. Pairwise chi-square tests for death/survival counts between resistant, intermediate susceptible and highly susceptible strains. (DOCX 55 kb) Additional file 2: Table S2. ANOVA analysis of main effects and interactions on body weight loss. Df: degrees of freedom; Sum Sq: sum of squares, Mean sq: mean sum of squares, Pr: p-value. ANOVA model: body weight loss ~ strain * sex * day; after deleting the non-significant three way interaction strain:day:sex. (DOCX 52 kb) Additional file 3: Table S3 Results from Tukey post-hoc HSD test for the interaction of strain:day of the ANOVA analysis from Table S2. (XLSX 83 kb) Additional file 4: Table S4. Post-hoc Tukey-HSD test of differences in viral loads between different groups of susceptible strains at day 3 p.i.. Results from ANOVA analysis (model: lg.viral.ld ~ suscept); lg.viral.ld: log2 viral load on day 3 p.i.; suscept: categories for resistant strains (resist), intermediate susceptible strains (int_susc) and highly susceptible strains (hlg_susc) are shown. Pairwise comparisons: int_susc-hlg_susc: intermediate susceptible versus highly susceptible strains, etc.; diff: difference between means of log2 viral load, p adj: adjusted p-value using Tukey HSD test. n = 36. (DOCX 52 kb) Additional file 5: Table S5. Post-hoc Tukey-HSD test of differences in viral load between different groups of susceptible strains at day 5 p.i. Results from ANOVA analysis (model: lg.viral.ld ~ suscept); lg.viral.ld: log2 viral load on day 5 p.i.; suscept: categories for resistant strains (resist), intermediate susceptible strains (int_susc) and highly susceptible strains (hlg_susc) are shown. Pairwise comparisons: int_susc-hlg_susc: intermediate susceptible versus highly susceptible strains, etc.; diff: difference between means of log2 viral load, p adj: adjusted p-value using Tukey HSD test. n = 41. (DOCX 52 kb) Additional file 6: Table S6. Average scoring of numbers of granulocytes and alveolar macrophages in C57BL/6 J, 129S1/SvImJ and CAST/EiJ lungs on day 5 after infection with H3N2 influenza virus from the histopathological study. (DOCX 53 kb) Additional file 7: Figure S1. Gene expression changes of inflammatory genes induced by influenza infection in 129S1/SvImJ, C56BL/6J, PWK/PhJ and CAST/EiJ lungs. Elevated gene expression levels of selected cytokines and chemokines in 129S1/SvImJ, C56BL/6J, PWK/PhJ and CAST/EiJ reflect influenza A infection. Expression values represent normalized log_2_ transformed signal intensities at different time points p.i. relative to expression levels in mock-infected control mice. (JPG 713 kb) Additional file 8: Figure S2. Gene expression changes of inflammatory genes induced by influenza infection in 129S1/SvImJ, C56BL/6J, PWK/PhJ and CAST/EiJ blood. Elevated gene expression levels of selected cytokines and chemokines in 129S1/SvImJ, C56BL/6J, PWK/PhJ and CAST/EiJ reflect influenza A infection. Expression values represent normalized log_2_ transformed signal intensities at different time points p.i. relative to expression levels in mock-infected control mice. (JPG 715 kb) Additional file 9: Figure S3. Gene expression changes of genes up-regulated in 129S1/SvImJ and C56BL/6J but not in CAST/EiJ in blood. Changes in the expression levels of probe sets that were up-regulated in 129S1/SvImJ and C56BL/6J but not in CAST/EiJ mice. Expression values represent normalized log_2_ transformed signal intensities at different time points p.i. relative to expression levels in mock-infected control mice. (JPG 395 kb) Additional file 10: Figure S4. Gene expression of selectins in lungs. Expression values represent normalized log_2_ transformed signal intensities at different time points p.i. relative to expression levels in mock-infected control mice. Probeset ID Sele: A_51_P455326 (JPG 652 kb) Additional file 11: Table S7. Marker descriptions of immune cells that were studied in the DCQ analysis. (DOCX 54 kb)
