# Supplementary material for: Influenza H3N2 infection of the collaborative cross founder strains reveals highly divergent host responses and identifies a unique phenotype in CAST/EiJ mice
Source: BMC Genomics. 2016 Feb 27;17:143. doi: 10.1186/s12864-016-2483-y (PMC4769537; doi:10.1186/s12864-016-2483-y)
Supplement: Additional file 1: Table S1. — Pairwise chi-square tests for death/survival counts between resistant, intermediate susceptible and highly susceptible strains. (DOCX 55 kb) [file 12864_2016_2483_MOESM1_ESM.docx]

## Table S1: Pairwise chi-square test for death / survival counts between resistant, intermediate and highly susceptible strains.

**Comparison 1**

Cell Contents

|-------------------------|

| Count |

| Expected Values |

| Chi-square contribution |

| Row Percent |

| Column Percent |

| Total Percent |

| Std Residual |

|-------------------------|

Total Observations in Table: 125

| dat3$status

dat3$sevty | 0 | 1 | Row Total |

-------------|-----------|-----------|-----------|

hlg_susc | 0 | 59 | 59 |

| 15.104 | 43.896 | |

| 15.104 | 5.197 | |

| 0.000% | 100.000% | 47.200% |

| 0.000% | 63.441% | |

| 0.000% | 47.200% | |

| -3.886 | 2.280 | |

-------------|-----------|-----------|-----------|

int_susc | 32 | 34 | 66 |

| 16.896 | 49.104 | |

| 13.502 | 4.646 | |

| 48.485% | 51.515% | 52.800% |

| 100.000% | 36.559% | |

| 25.600% | 27.200% | |

| 3.675 | -2.155 | |

-------------|-----------|-----------|-----------|

Column Total | 32 | 93 | 125 |

| 25.600% | 74.400% | |

-------------|-----------|-----------|-----------|

Statistics for All Table Factors

Pearson's Chi-squared test

------------------------------------------------------------

Chi^2 = 38.44901 d.f. = 1 p = 5.620319e-10

Fisher's Exact Test for Count Data

------------------------------------------------------------

Sample estimate odds ratio: 0

Alternative hypothesis: true odds ratio is not equal to 1

p = 1.730967e-11

95% confidence interval: 0 0.0779173

Alternative hypothesis: true odds ratio is less than 1

p = 1.132874e-11

95% confidence interval: 0 0.06180243

Alternative hypothesis: true odds ratio is greater than 1

p = 1

95% confidence interval: 0 Inf

**Comparison 2**

Cell Contents

|-------------------------|

| Count |

| Expected Values |

| Chi-square contribution |

| Row Percent |

| Column Percent |

| Total Percent |

| Std Residual |

|-------------------------|

Total Observations in Table: 107

| dat3$status

dat3$sevty | 0 | 1 | Row Total |

-------------|-----------|-----------|-----------|

int_susc | 32 | 34 | 66 |

| 45.028 | 20.972 | |

| 3.769 | 8.093 | |

| 48.485% | 51.515% | 61.682% |

| 43.836% | 100.000% | |

| 29.907% | 31.776% | |

| -1.942 | 2.845 | |

-------------|-----------|-----------|-----------|

resist | 41 | 0 | 41 |

| 27.972 | 13.028 | |

| 6.068 | 13.028 | |

| 100.000% | 0.000% | 38.318% |

| 56.164% | 0.000% | |

| 38.318% | 0.000% | |

| 2.463 | -3.609 | |

-------------|-----------|-----------|-----------|

Column Total | 73 | 34 | 107 |

| 68.224% | 31.776% | |

-------------|-----------|-----------|-----------|

Statistics for All Table Factors

Pearson's Chi-squared test

------------------------------------------------------------

Chi^2 = 30.95849 d.f. = 1 p = 2.636066e-08

Fisher's Exact Test for Count Data

------------------------------------------------------------

Sample estimate odds ratio: 0

Alternative hypothesis: true odds ratio is not equal to 1

p = 9.340624e-10

95% confidence interval: 0 0.100639

Alternative hypothesis: true odds ratio is less than 1

p = 7.539568e-10

95% confidence interval: 0 0.0796327

Alternative hypothesis: true odds ratio is greater than 1

p = 1

95% confidence interval: 0 Inf

**Comparison 3**

Cell Contents

|-------------------------|

| Count |

| Expected Values |

| Chi-square contribution |

| Row Percent |

| Column Percent |

| Total Percent |

| Std Residual |

|-------------------------|

Total Observations in Table: 100

| dat3$status

dat3$sevty | 0 | 1 | Row Total |

-------------|-----------|-----------|-----------|

hlg_susc | 0 | 59 | 59 |

| 24.190 | 34.810 | |

| 24.190 | 16.810 | |

| 0.000% | 100.000% | 59.000% |

| 0.000% | 100.000% | |

| 0.000% | 59.000% | |

| -4.918 | 4.100 | |

-------------|-----------|-----------|-----------|

resist | 41 | 0 | 41 |

| 16.810 | 24.190 | |

| 34.810 | 24.190 | |

| 100.000% | 0.000% | 41.000% |

| 100.000% | 0.000% | |

| 41.000% | 0.000% | |

| 5.900 | -4.918 | |

-------------|-----------|-----------|-----------|

Column Total | 41 | 59 | 100 |

| 41.000% | 59.000% | |

-------------|-----------|-----------|-----------|

Statistics for All Table Factors

Pearson's Chi-squared test

------------------------------------------------------------

Chi^2 = 100 d.f. = 1 p = 1.523971e-23

Fisher's Exact Test for Count Data

------------------------------------------------------------

Sample estimate odds ratio: 0

Alternative hypothesis: true odds ratio is not equal to 1

p = 4.971058e-29

95% confidence interval: 0 0.003171025

Alternative hypothesis: true odds ratio is less than 1

p = 4.971058e-29

95% confidence interval: 0 0.002308842

Alternative hypothesis: true odds ratio is greater than 1

p = 1

95% confidence interval: 0 Inf
