# Supplementary material for: Influenza H3N2 infection of the collaborative cross founder strains reveals highly divergent host responses and identifies a unique phenotype in CAST/EiJ mice
Source: BMC Genomics. 2016 Feb 27;17:143. doi: 10.1186/s12864-016-2483-y (PMC4769537; doi:10.1186/s12864-016-2483-y)
Supplement: Additional file 2: Table S2. — ANOVA analysis of main effects and interactions on body weight loss. Df: degrees of freedom; Sum Sq: sum of squares, Mean sq: mean sum of squares, Pr: p-value. ANOVA model: body weight loss ~ strain * sex * day; after deleting the non-significant three way interaction strain:day:sex. (DOCX 52 kb) [file 12864_2016_2483_MOESM2_ESM.docx]

## Table S2: ANOVA summary of main effects and interactions (reduced model without non-significant interactions) on body weight loss.

Df Sum Sq Mean Sq F value Pr(>F)

strain 7 13924 1989 98.358 < 2e-16

day 6 27556 4593 227.089 < 2e-16

sex 1 2168 2168 107.216 < 2e-16

strain:day 42 14632 348 17.226 < 2e-16

strain:sex 7 1190 170 8.403 4.99e-10

day:sex 6 629 105 5.181 2.89e-05

Residuals 1070 21640 20

Df: degrees of freedom; Sum Sq: sum of squares, Mean sq: mean sum of squares, Pr: p-value. ANOVA model: body weight loss ~ strain * sex * day; after deleting the non-significant three way interaction strain:day:sex.
