# Supplementary material for: Influenza H3N2 infection of the collaborative cross founder strains reveals highly divergent host responses and identifies a unique phenotype in CAST/EiJ mice
Source: BMC Genomics. 2016 Feb 27;17:143. doi: 10.1186/s12864-016-2483-y (PMC4769537; doi:10.1186/s12864-016-2483-y)
Supplement: Additional file 5: Table S5. — Post-hoc Tukey-HSD test of differences in viral load between different groups of susceptible strains at day 5 p.i. Results from ANOVA analysis (model: lg.viral.ld ~ suscept); lg.viral.ld: log2 viral load on day 5 p.i.; suscept: categories for resistant strains (resist), intermediate susceptible strains (int_susc) and highly susceptible strains (hlg_susc) are shown. Pairwise comparisons: int_susc-hlg_susc: intermediate susceptible versus highly susceptible strains, etc.; diff: difference between means of log2 viral load, p adj: adjusted p-value using Tukey HSD test. n = 41. (DOCX 52 kb) [file 12864_2016_2483_MOESM5_ESM.docx]

## Table S5: Post-hoc Tukey-HSD test of differences in viral load between different susceptible strains at day 5 p.i..

diff p adj

int_susc-hlg_susc -0.1469787 9.829827e-01

resist-hlg_susc -5.1911874 1.575851e-05

resist-int_susc -5.0442087 2.517064e-05

Results from ANOVA analysis (model: lg.viral.ld ~ suscept); lg.viral.ld: log2 viral load on day 5 p.i.; suscept: categories for resistant strains (resist), intermediate susceptible strains (int_susc) and highly susceptible strains (hlg_susc). Pairwise comparisons: int_susc-hlg_susc: intermediate susceptible versus highly susceptible strains, etc.; diff: difference between means of log2 viral load, p adj: adjusted p-value using Tukey HSD test. n=41.
