# Supplementary material for: Influenza H3N2 infection of the collaborative cross founder strains reveals highly divergent host responses and identifies a unique phenotype in CAST/EiJ mice
Source: BMC Genomics. 2016 Feb 27;17:143. doi: 10.1186/s12864-016-2483-y (PMC4769537; doi:10.1186/s12864-016-2483-y)
Supplement: Additional file 6: Table S6. — Average scoring of numbers of granulocytes and alveolar macrophages in C57BL/6 J, 129S1/SvImJ and CAST/EiJ lungs on day 5 after infection with H3N2 influenza virus from the histopathological study. (DOCX 53 kb) [file 12864_2016_2483_MOESM6_ESM.docx]

## Table S6: Average scoring of numbers of granulocytes and alveolar macrophages in C57BL/6J, 129S1/SvImJ and CAST/EiJ lungs on day 5 after infection with H3N2 influenza virus.

| Strain | Group | Granulocytes | sd | Alveolar macrophages | sd |
| --- | --- | --- | --- | --- | --- |
| C57BL/6J | mock d3 | 19.7 | 21.5 | 76.7 | 16.8 |
|  | d5 | 432.7 | 250.2 | 110.7 | 36.6 |
| 129S1/SvImJ | mock d3 | 53.3 | 18.6 | 111.7 | 32.3 |
|  | d5 | 379.3 | 228.2 | 99.7 | 73.2 |
| CAST/EiJ | mock d3 | 126.0 | 50.7 | 26.7 | 12.7 |
|  | d5 | 183.3 | 53.5 | 137.0 | 33.18 |
