# Supplementary figures and images for: Influenza H3N2 infection of the collaborative cross founder strains reveals highly divergent host responses and identifies a unique phenotype in CAST/EiJ mice
Source: BMC Genomics. 2016 Feb 27;17:143. doi: 10.1186/s12864-016-2483-y (PMC4769537; doi:10.1186/s12864-016-2483-y)

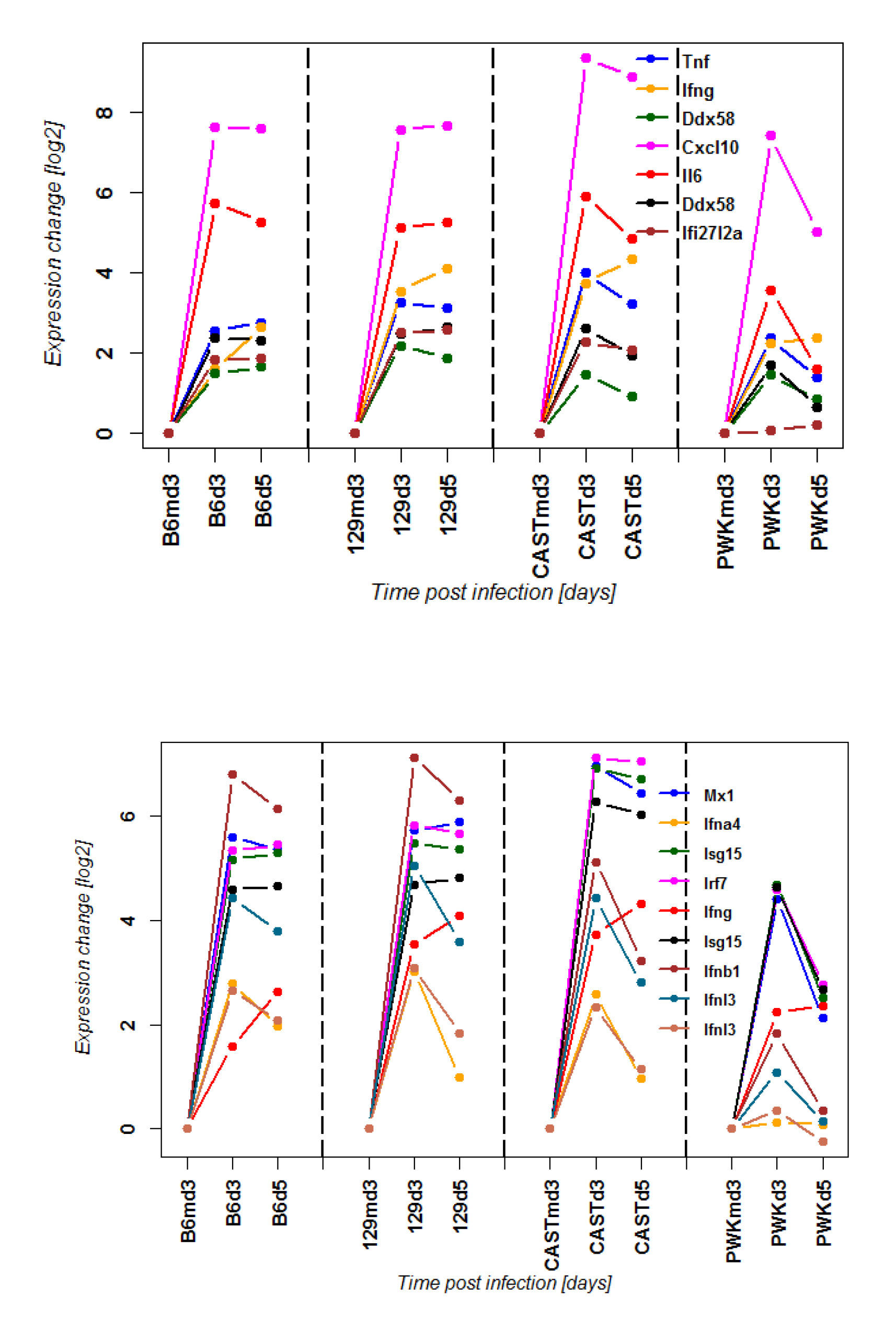

Supplement: Additional file 7: Figure S1. — Gene expression changes of inflammatory genes induced by influenza infection in 129S1/SvImJ, C56BL/6J, PWK/PhJ and CAST/EiJ lungs. Elevated gene expression levels of selected cytokines and chemokines in 129S1/SvImJ, C56BL/6J, PWK/PhJ and CAST/EiJ reflect influenza A infection. Expression values represent normalized log2 transformed signal intensities at different time points p.i. relative to expression levels in mock-infected control mice. (JPG 713 kb) [file 12864_2016_2483_MOESM7_ESM.jpg]

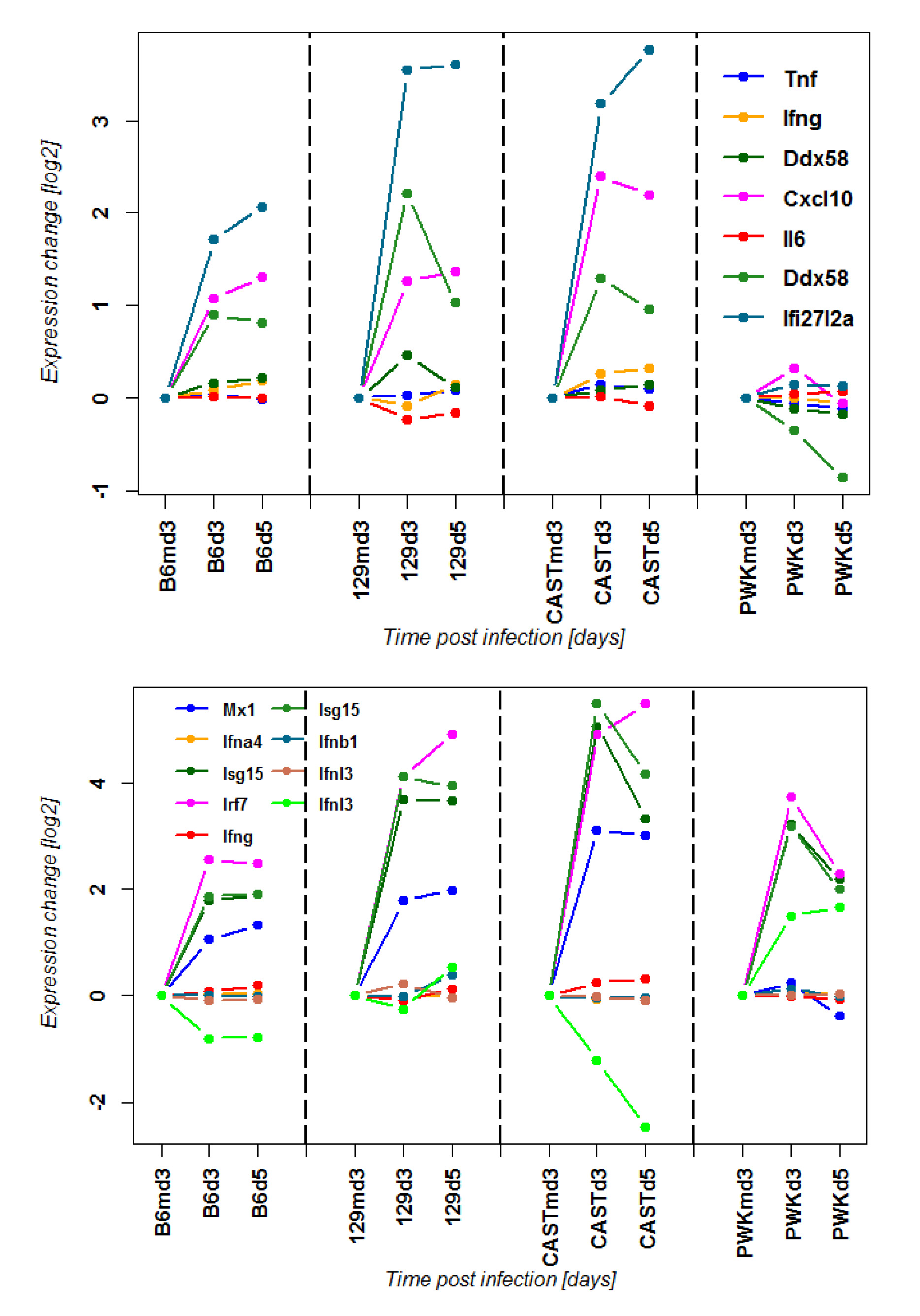

Supplement: Additional file 8: Figure S2. — Gene expression changes of inflammatory genes induced by influenza infection in 129S1/SvImJ, C56BL/6J, PWK/PhJ and CAST/EiJ blood. Elevated gene expression levels of selected cytokines and chemokines in 129S1/SvImJ, C56BL/6J, PWK/PhJ and CAST/EiJ reflect influenza A infection. Expression values represent normalized log2 transformed signal intensities at different time points p.i. relative to expression levels in mock-infected control mice. (JPG 715 kb) [file 12864_2016_2483_MOESM8_ESM.jpg]

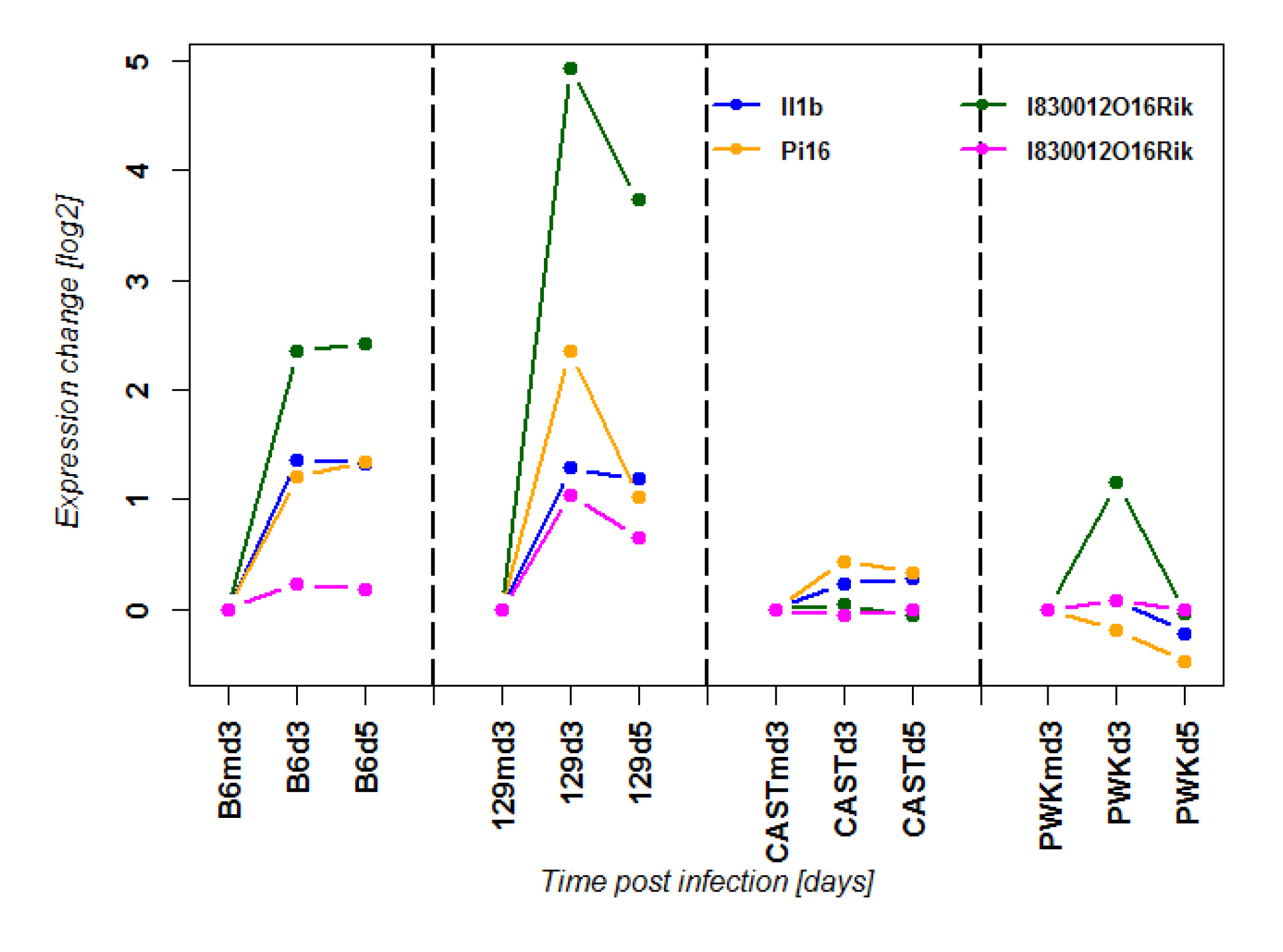

Supplement: Additional file 9: Figure S3. — Gene expression changes of genes up-regulated in 129S1/SvImJ and C56BL/6J but not in CAST/EiJ in blood. Changes in the expression levels of probe sets that were up-regulated in 129S1/SvImJ and C56BL/6J but not in CAST/EiJ mice. Expression values represent normalized log2 transformed signal intensities at different time points p.i. relative to expression levels in mock-infected control mice. (JPG 395 kb) [file 12864_2016_2483_MOESM9_ESM.jpg]

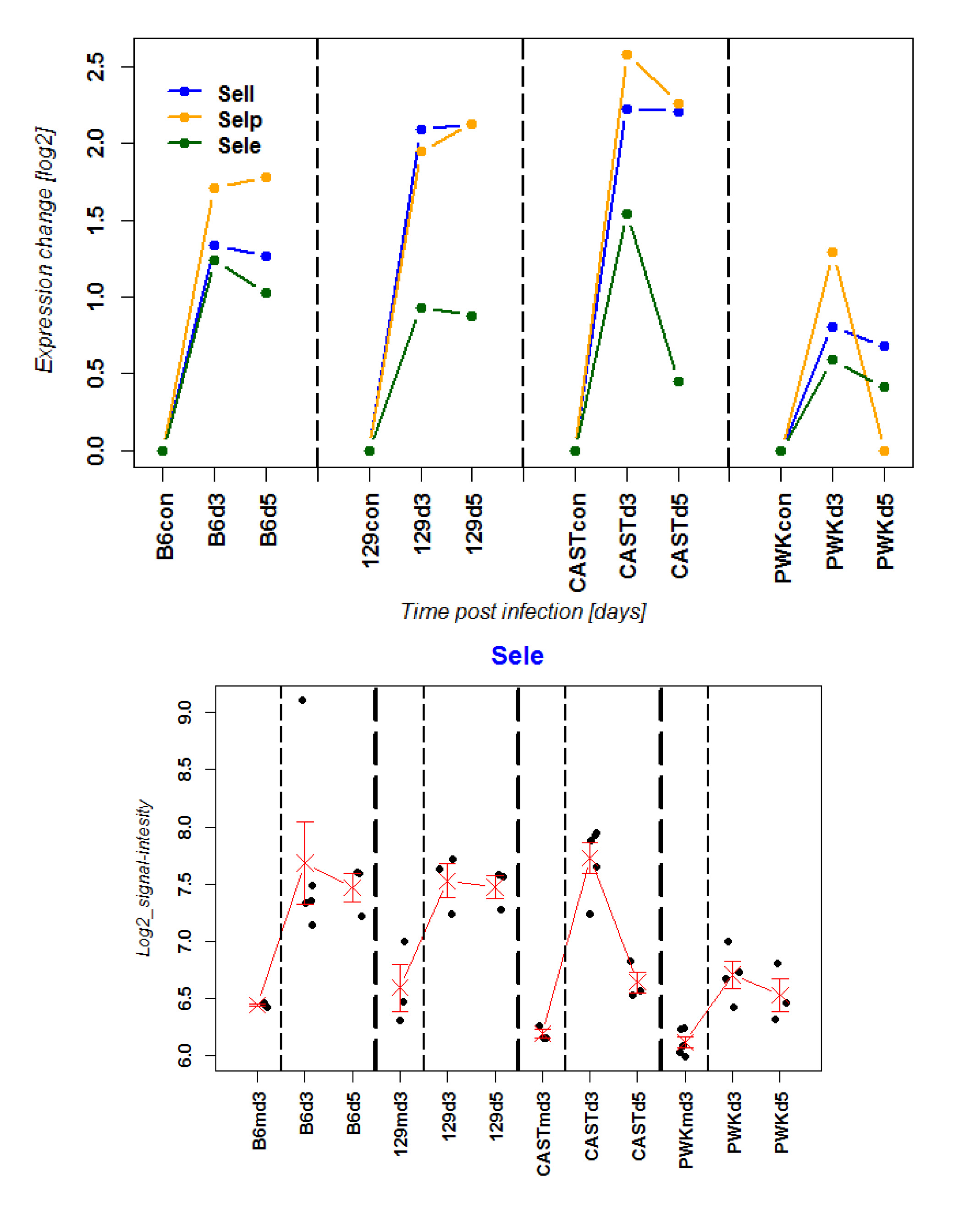

Supplement: Additional file 10: Figure S4. — Gene expression of selectins in lungs. Expression values represent normalized log2 transformed signal intensities at different time points p.i. relative to expression levels in mock-infected control mice. Probeset ID Sele: A_51_P455326 (JPG 652 kb) [file 12864_2016_2483_MOESM10_ESM.jpg]
