# Supplementary material for: Influenza H3N2 infection of the collaborative cross founder strains reveals highly divergent host responses and identifies a unique phenotype in CAST/EiJ mice
Source: BMC Genomics. 2016 Feb 27;17:143. doi: 10.1186/s12864-016-2483-y (PMC4769537; doi:10.1186/s12864-016-2483-y)
Supplement: Additional file 11: Table S7. — Marker descriptions of immune cells that were studied in the DCQ analysis. (DOCX 54 kb) [file 12864_2016_2483_MOESM11_ESM.docx]

## Table S7: DCQ immune cell populations.

| **Short name** | **Full name** | **Phenotype** |
| --- | --- | --- |
| GN.BM | Neutrophils | CD11b+ Gr1+ 7/4hi |
| GN.Arth.BM | Neutrophils, Arthritic | CD11b+ Ly6-G+ |
| GN.Bl | Neutrophils from Blood | CD11b+ Ly6-G+ |
| GN.Thio.PC | Neutrophils, Thioglycolate | CD11b+ Ly6-G+ |
| GN.UrAc.PC | Neutrophils, Uric Acid | CD11b+ Ly6-G+ |
| Mo.6C-IIint.Bl | Nonclassical Monocytes, MHCIIint | B220- CD43+ CD115+ Ly-6C- MHCIIint |
| Mo.6C-II+.Bl | Nonclassical Monocytes, MHCIIhi | CD43- CD115+ Ly-6C- MHCIIhi |
| Mo.6C+II+.Bl | Classical Monocytes, MHCII+ | CD43- CD115+ Ly-6C+ MHCII+ |
| Mo.6C+II-.LN | Classical Monocytes, MHCII- | B220- MHCII- F4/80+ CD11c- CD11bhi NK1.1- Gr1+ |
| MF.103-11b+24-.Lu | Lung CD11b+24- macrophage | MHCII+ CD11c+ CD11bhi CD103- CD24- |
| DC.103-11b+F4/80lo.Kd | Kidney CD11b+ F4/80lo dendritic cells (P7, CD11b hi) | CD45+ CD3- B220- NKp46- CD11c+ MHCII+ CD11b+ F4/80lo |
| Mo.6C+II+.Bl | Classical Monocytes, MHCII+ | CD43- CD115+ Ly-6C+ MHCII+ |
| Mo.6C+II-.LN | Classical Monocytes, MHCII- | B220- MHCII- F4/80+ CD11c- CD11bhi NK1.1- Gr1+ |
| Mo.6C+II-.BM | Classical Monocytes | B220- CD3- CD115+ Ly-6C+ MHCII- |
| Mo.6C-II-.Bl | Nonclassical Monocytes, MHCII- | B220- CD43+ CD115+ Ly-6C- MHCII |
| MF.Medl.SLN | NA | CD169 (3D6.112) – FITC; Ly6G (1AE) – PE; CD103 (2E7) – PE; SiglecF(E50-2440) – PE; CD90.2 (30-H12) – biotin; B220 (RA3-6B2) – biotin; Streptavidin – PerCPCy5.5; CD11c (N418) – PECy7; CD11b (M1/70) – Alexa-eFluor780; F4/80 (CI:A3-1) – AlexaFluor 647 |
| DC.8+.Sp | Spleen CD8+ dendritic cell | CD11c+ CD8a+ CD4- CD11b- |
| DC.103+11b-.Lv | Liver CD103+ dendritic cells | CD45+ CD11c+ MHCII+ CD11blo CD103+ |
| DC.8+.SLN | Skin draining LN CD8+ dendritic cell | CD11c+ CD8a+ CD4- CD11b- |
| DC.8+.MLN | Mesenteric LN CD8+ dendritic cell | CD11c+ CD8a+ CD4- CD11b- |
